# Predicting acute clinical deterioration with interpretable machine learning to support emergency care decision making

**DOI:** 10.1038/s41598-023-40661-0

**Published:** 2023-08-21

**Authors:** Stelios Boulitsakis Logothetis, Darren Green, Mark Holland, Noura Al Moubayed

**Affiliations:** 1 Department of Computer Science, University of Durham, Durham, UK; 2 Department of Renal Medicine, Northern Care Alliance NHS Foundation Trust, Manchester, UK; 3 Division of Cardiovascular Sciences, University of Manchester, Manchester, UK; 4 School of Clinical and Biomedical Sciences, University of Bolton, Bolton, UK; 5 Evergreen Life Ltd, Manchester, UK

**Keywords:** Computational biology and bioinformatics, Machine learning

## Abstract

The emergency department (ED) is a fast-paced environment responsible for large volumes of patients with varied disease acuity. Operational pressures on EDs are increasing, which creates the imperative to efficiently identify patients at imminent risk of acute deterioration. The aim of this study is to systematically compare the performance of machine learning algorithms based on logistic regression, gradient boosted decision trees, and support vector machines for predicting imminent clinical deterioration for patients based on cross-sectional patient data extracted from electronic patient records (EPR) at the point of entry to the hospital. We apply state-of-the-art machine learning methods to predict early patient deterioration, based on their first recorded vital signs, observations, laboratory results, and other predictors documented in the EPR. Clinical deterioration in this study is measured by in-hospital mortality and/or admission to critical care. We build on prior work by incorporating interpretable machine learning and fairness-aware modelling, and use a dataset comprising 118, 886 unplanned admissions to Salford Royal Hospital, UK, to systematically compare model variations for predicting mortality and critical care utilisation within 24 hours of admission. We compare model performance to the National Early Warning Score 2 (NEWS2) and yield up to a 0.366 increase in average precision, up to a $21.16\%$ reduction in daily alert rate, and a median 0.599 reduction in differential bias amplification across the protected demographics of age and sex. We use Shapely Additive exPlanations to justify the models’ outputs, verify that the captured data associations align with domain knowledge, and pair predictions with the causal context of each patient’s most influential characteristics. Introducing our modelling to clinical practice has the potential to reduce alert fatigue and identify high-risk patients with a lower NEWS2 that might be missed currently, but further work is needed to trial the models in clinical practice. We encourage future research to follow a systematised approach to data-driven risk modelling to obtain clinically applicable support tools.

## Introduction

When patients deteriorate, care providers must be able to recognise their worsening condition immediately and intervene accordingly^1^. Delayed identification of deterioration is associated with preventable hospital deaths^2^, while delaying the transfer of critically ill patients to intensive care puts them at higher risk of morbidity and mortality^3^. The importance of timely identification and appropriate response to clinical instability has motivated the development of ’track-and-trigger’ systems. These systems tie clinical observations that are antecedent to patient deterioration with recommended interventions to be executed by care staff or dedicated response teams as part of a rapid response system^4^. In the United Kingdom, this system is recommended by both National Institute for Health and Care Excellence (NICE) and the Royal College of Physicians (RCP) to monitor all adult patients in acute hospital settings^5,6^.

In most cases, acute clinical instability and deterioration are preceded by abnormal vital signs^7^, therefore standard practice in acute secondary care settings is to monitor patients using basic homeostatic measures, which include heart rate, blood pressure, inspired oxygen, oxygen saturation, temperature, and level of consciousness^8^. To assist this process, weighted aggregate scores of these measures, known as Early Warning Scores (EWS), have been developed to characterise the patient’s acuity^9^. These scores can act as the afferent component of a rapid response system, tying them to an escalation protocol or a set of recommended clinical interventions^4^.

Historically, data pertaining to an EWS were manually recorded and tallied on paper charts. As such, they often fell short of including the full breadth and variety of available predictive information^10^. The gradual phasing-out of bedside paper charts has brought the transition to digital EWS solutions that draw patient data in real-time from Electronic Patient Records (EPR). Beyond digitising conventional EWS, EPR systems collate comprehensive patient data, which can be used to improve performance and clinical utility^11^. In particular, the large volume of available data makes it feasible to develop a purely or partly data-driven solution using machine learning. AI-based systems have already demonstrated suitability for assisting in medical imaging tasks, which makes AI-powered prognostic modelling a key research area of interest^12^. Our study concentrates on analysing EPR data to model clinical risk, as we use machine learning methods to potentially identify acute clinical deterioration in patients presenting to the Emergency Department (ED).

Prior work has used machine learning to model inpatient admission, deterioration, critical care admission, cardiac arrest, and mortality, among other outcomes^13^. In a systematic review of studies published from 2009–2017, Goldstein et al. identified 107 applications of EPR data to training statistical and ML models^11^. Recently, Klug et al.^14^ used gradient-boosted decision trees (GBDT) on a single-centre cohort of approximately 800,000 ED episodes to predict short-term mortality risk and achieved improved performance over severity scores such as the Shock Index^15^. Romero et al.^16^ developed a gradient-boosting machine (GBM) model for use as an EWS and demonstrated superior performance compared to the National Early Warning Score 2 (NEWS2)^6^. Finally, Fernandes et al.^17^ investigated the predictive value of ED patients’ presenting complaints compared to vital signs and other measurements. They used natural language processing (vectorisation with TF-IDF normalisation) to encode presenting complaints and trained models on a cohort of approximately 235, 000 patients to predict mortality or cardiac arrest. Their findings showed improved predictive performance and calibration when including the chief complaint as a predictor.

This study applies state-of-the-art methods from contemporary machine learning practice to estimate risk of deterioration for acute medical patients in the ED. We bring together findings from prior studies to improve the differentiation of at-risk patients and address challenges that are prerequisites to clinical deployment for a proposed solution. The ED is a fast-paced environment that treats a large volume of patients with varied acuity and is responsible for their initial assessment and clinical management^18^. Operational pressures in EDs are steadily increasing^19^, creating an imperative to differentiate the patients with the highest risk efficiently. In our study setting, ’obvious cases’ of imminent critical deterioration usually bypass the acute medical team and are escalated immediately. By elimination, the remaining patients are ’less obvious’ cases and thus have a greater need for decision support. Conventional, general-purpose EWS are not optimised for specific patient populations or contexts, while ’off-the-shelf’ EWS, such as the NEWS2, have variable performance^20^. Recent work argues in favour of centre-specific, locally tailored scores and risk models^21,22^; data-driven solutions deployable at scale can fulfil this role.

Our outcome of interest is a composite of in-hospital mortality and admission to critical care to represent severe and time-sensitive medical conditions requiring intervention. We ensure the models’ outputted probabilities are well-calibrated and reliable to fit into existing frameworks for assessing clinical utility^23^. Rather than prescribe a specific threshold for classifying high-risk cases, we measure our models’ discriminative skill across sensitivities via precision-recall curves and through their daily alert rate, which expresses how they would operate when deployed. We compare our performance against NEWS2, the preferred EWS in the United Kingdom^24^.

An extant practical challenge we address is models not generalising to new application environments due to structural differences compared to the development environment^25,26^. Solutions with rigid data requirements unrealistically require providers to conform to a specific pattern of testing or treatment to produce all the requisite data correctly^25^. To avoid making assumptions about data availability or its collection context (such as timing, reliability, or frequency), we conduct experiments using different sets of predictive features that providers might generate under their unique clinical workflow. Starting with vital signs, we gradually construct models with finer information, including manual observations, laboratory results, clinical notes, and service utilisation, to reveal the most influential features.

A further barrier is a requirement for models supporting the clinical workflow to be transparent, safe, fair, and traceable in their decision-making process^27,28^. Machine learning models have conventionally operated as ’black boxes’^29^, obscuring their internal reasoning and biases^28,30^. Advances in interpretable machine learning and fairness-aware modelling allow us to address this. We incorporate methods from the fair machine learning literature^31,32^ into our evaluation framework to ensure our constructed models do not exhibit unfair bias against individuals or protected demographic groups. Then, we utilise Shapely Additive exPlanations^33^, a recently popularised model-agnostic framework for interpreting predictive models, to produce justifications for our models’ risk predictions on the individual patient level. These justifications reveal the best-performing models’ internal reasoning and allow us to examine and validate the relationships between the significant predictors and the outcome. In addition to predicting a patient’s risk, our interpretable models can justify their prediction to the user by isolating the relevant characteristics of the patient that led them to that result^33,34^.

The aim of this study is to systematically compare the performance of various learning algorithms based on logistic regression (LR), gradient-boosted decision trees (GBDT), and support vector machines (SVM) for predicting imminent clinical deterioration for patients admitted to the emergency admissions unit based on cross-sectional patient data extracted from EPR at the point of entry to hospital. We compare predictive performance to NEWS2. While this study is not designed to test novel predictors of acute deterioration, using interpretable machine learning to model multiple patient-related variables does allow a comparison of these variables and their contribution to identifying adverse outcomes.

## Results

Our selected data comprised 118,886 presentations to the Emergency Admissions Unit (EAU) at Salford Royal Hospital, Manchester, UK, corresponding to 61,611 distinct patients over the study period of January 2015 to March 2022. We identified 8286 critical deterioration events, of which 2885 occurred within 24 hours after admission. Table 1 summarises the dataset and presents the stratification of samples across the three data subsets we used in our analysis: we partitioned the samples chronologically 2:1 into a model development set and a validation set and additionally extracted two subsets of the validation set. The ’unseen’ validation subset excludes the 8054 patients ($13.07\%$, making up $42.14\%$ of the validation set’s records) that had prior admission records in the training set, and the ’pre-Covid’ subset only includes validation admissions that occurred prior to March 1st, 2020. The rates of critical care admission, mortality, and composite critical deterioration were uniform across the chronological split.

**Table 1:** Summary statistics of the study sample.

Group	Variable	Total	Train	Valid (complete)	Valid (unseen)	Valid (pre-covid)
Episode	Records	118,886	79,653	39,233	22,701	9613
	Patients	61,611	44,323	25,342	17,288	7672
	LOS (days)	2.29 (0.66–7.14)	2.06 (0.63–6.67)	2.93 (0.73–8.57)	2.27 (0.58–7.59)	2.85 (0.73–7.87)
Outcomes	30-day mortality	3908 (3.29%)	2545 (3.20%)	1363 (3.47%)	685 (3.02%)	313 (3.26%)
	Critical care	3982 (3.35%)	2794 (3.51%)	1188 (3.03%)	717 (3.16%)	347 (3.61%)
	Critical event	2885 (2.43%)	2008 (2.52%)	877 (2.24%)	519 (2.29%)	256 (2.66%)
	In-hospital mortality	5092 (4.28%)	3213 (4.03%)	1879 (4.79%)	959 (4.22%)	363 (3.78%)
Vitals	AVCPU-A	117,324 (98.69%)	78,563 (98.63%)	38,761 (98.80%)	22,449 (98.89%)	9492 (98.74%)
	Assisted breathing	12,116 (10.19%)	7785 (9.77%)	4331 (11.04%)	2254 (9.93%)	1053 (10.95%)
	NEWS2	1 (0–2)	1 (0–2)	1 (0–2)	1 (0–2)	1 (0–2)
	Pulse (beats/min)	80 (70–90)	80 (70–90)	80 (70–90)	80 (70–90)	80 (70–90)
	RR (breaths/min)	17 (16–18)	17 (16–18)	18 (16–18)	17 (16–18)	17 (16–18)
	SpO_2_ (%)	97 (96–98)	97 (96–98)	97 (96–98)	97 (96–98)	97 (96–98)
	Systolic BP (mmHg)	124 (113–139)	122 (112–138)	125 (114–140)	125 (114–140)	124 (114–138)
	Temperature (oC)	36.70 (36.40–37)	36.70 (36.40–37)	36.70 (36.40–37)	36.70 (36.40–37)	36.70 (36.40–37)
Supplemental obs. & phenotype	Age (years)	69 (50–82)	69 (50–82)	69 (50–81)	64 (44–79)	69 (49–82)
	Diastolic BP (mmHg)	70 (60–80)	70 (60–78)	70 (62–80)	70 (62–80)	70 (60–79)
	Female	62,355 (52.45%)	42,029 (52.77%)	20,326 (51.81%)	11,395 (50.20%)	5021 (52.23%)
	FiO_2_ (%)	0 (0–0)	0 (0–0)	0 (0–0)	0 (0–0)	0 (0–0)
	Lying down*	55,950 (47.06%)	35,750 (44.88%)	20,200 (51.49%)	11,310 (49.82%)	4666 (48.54%)
	Nausea	1911 (1.61%)	1390 (1.75%)	521 (1.33%)	274 (1.21%)	116 (1.21%)
	Pain	18,201.0 (15.31%)	13,117.0 (16.47%)	5084.0 (12.96%)	3149.0 (13.87%)	1092.0 (11.36%)
	Vomiting	598 (0.50%)	411 (0.52%)	187 (0.48%)	105 (0.46%)	35 (0.36%)
Labs	Creatinine (mmol/L)	78 (63–104)	77 (62–102)	79 (64–105)	77 (63–100)	79 (63–104)
	Haemoglobin (g/L)	130 (115–143)	130 (115–143)	130 (115–143)	132 (117–145)	130 (115–143)
	Potassium (mEg/L)	4.20 (3.90–4.50)	4.20 (3.90–4.50)	4.20 (3.90–4.50)	4.20 (3.90–4.50)	4.20 (3.90–4.50)
	Sodium (mmol/L)	138 (135-140)	138 (135–140)	138 (135-140)	138 (135-140)	138 (135–140)
	Urea (mmol/L)	6.30 (4.60–9.50)	6.30 (4.60–9.30)	6.40 (4.60–9.60)	6 (4.50–8.90)	6.20 (4.50–9.30)
Service utilisation	Readmission	14601 (12.28%)	10,278 (12.90%)	4323 (11.02%)	1719 (7.57%)	1119 (11.64%)
	SDEC	27,979 (23.53%)	20,488 (25.72%)	7491 (19.09%)	5332 (23.49%)	2063 (21.46%)

Numerical patient characteristics of EAU admissions, chronologically partitioned into training and validation sets. “Test (Unseen)” corresponds to the chronologically split validation set but excluding patients who had any prior admissions in the training set. Binary variables are reported as “number of positives (%)”, while numerical variables are reported as quartiles.

*Lying down refers to the patients’ position when their blood pressure was recorded. By default, patients not lying down are assumed to be sitting.

**Figure 1:**
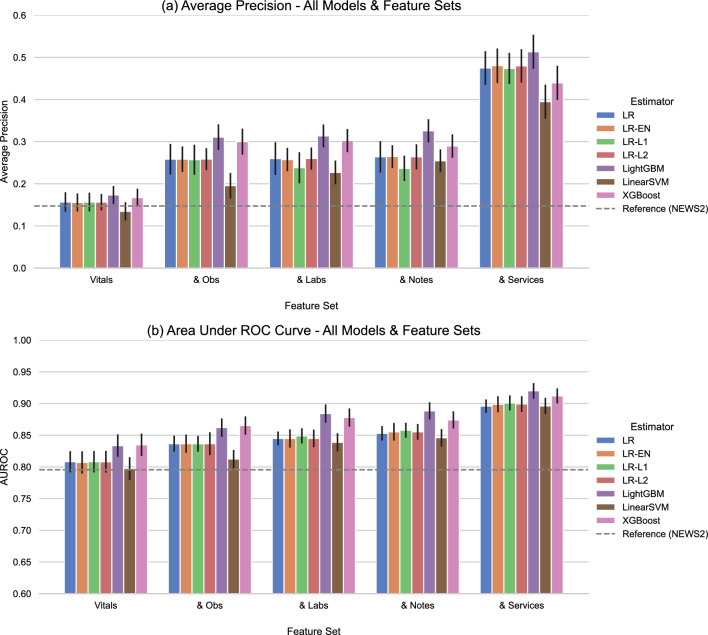
Average precision (**a**) and Area under receiver operating curve (**b**) achieved by the best predictive models per learning algorithm across tested sets of data features. Each group corresponds to independent models trained with the indicated feature set concatenated to all the previous feature sets to its right. The error bars represent $95\%$ bootstrapped confidence intervals. Obs: Supplemental observations & phenotype, Labs: Laboratory results, Notes: Clinical notes, Services: Triage & service utilisation. We detail the contents of the feature sets in Methods Table 4.

We compared numerous modelling pipeline variations as described in the "Methods" section. From this comparison, we identified LightGBM, a variant of GBDT, as the best-performing learning algorithm overall and logistic regression with L2 penalty (LR-L2) as the best linear model. We summarise their performance in Table 2. Figure 1 compares the average precision (AP) and area under the receiver operating curve (AUROC) of the best predictive models across classifier types on the complete validation set against the measured performance of the reference model (NEWS2) on this patient cohort. The groups in each plot correspond to incrementally augmenting the training data - the leftmost groups of each section present models using only vital signs as predictors, and subsequent groups give the results when we concatenated the indicated feature set (as described in Methods Table 4) to the previous training inputs. We test these sets of features in order of ’centre-specificity’, so that the most clinically standardised predictors, such as vital signs, are considered first. We provide the actual measurements with bootstrapped confidence intervals and the performance on the ’unseen’ and ’pre-covid’ validation sets in Supplementary Tables 4 and 5.

**Figure 2:**
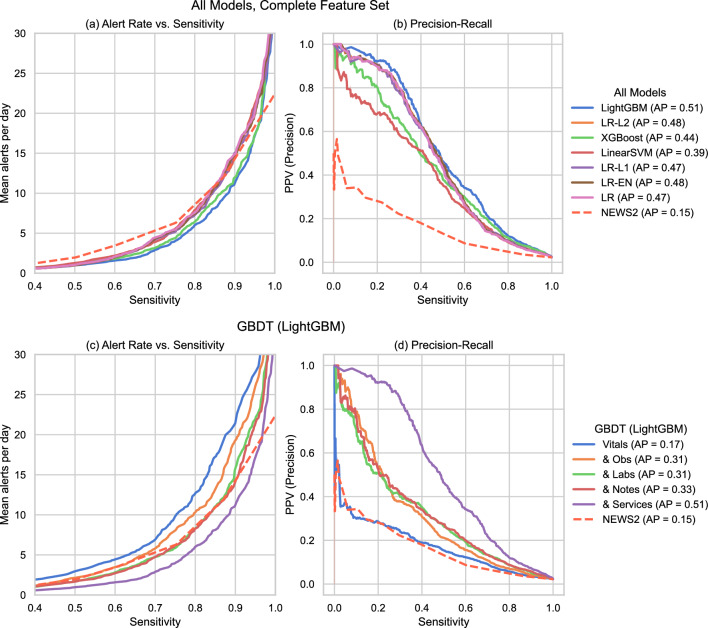
Alert Rate vs Sensitivity (**a,c**) and Precision-Recall curves (**b,d**). Top Row (**a,b**): All learning algorithms trained on the complete feature set (equiv. “& Services”). Bottom Row (**c,d**): LightGBM (GBDT) across feature sets (concatenated incrementally). In (**a,c**), the Alert Rate curve plots the arithmetic mean of daily positive predictions (alerts) across the validation period for a given sensitivity value (y-axis) against that sensitivity value (x-axis). The point where two lines intersect corresponds to the maximum achievable sensitivity for which the model with the lower line maintains a lower daily alert rate than the model with the upper line. In (**b,d**), the Precision-Recall (PR) curve presents the positive predictive value (PPV, or precision) on the y-axis against sensitivity on the x-axis. On the PR curve, an unskilled model giving random outputs would yield a horizontal line at $y=P/(P+N)$, where *P* and *N* are the numbers of positive and negative samples in the data, respectively, while a theoretical ‘perfect’ model would yield a single point (1, 1) in the upper-right corner of the plot. The curves are plotted from each model’s outputted predictions for the complete validation set.

**Table 2:** Summary of model performance.

Metric	Estimator	Dataset	Vitals	& Obs	& Labs	& Notes	& Services
AP	LR-L2	Complete	0.156	0.259	0.260	0.264	0.480
		Pre-covid	0.163	0.262	0.257	0.266	0.535
		Unseen	0.172	0.311	0.302	0.311	0.489
	LightGBM	Complete	0.173	0.311	0.314	0.326	0.513
		Pre-covid	0.194	0.322	0.318	0.355	0.571
		Unseen	0.198	0.371	0.364	0.373	0.525
AUROC	LR-L2	Complete	0.808	0.837	0.845	0.855	0.899
		Pre-covid	0.800	0.828	0.829	0.845	0.903
		Unseen	0.820	0.845	0.852	0.858	0.901
	LightGBM	Complete	0.834	0.862	0.884	0.888	0.920
		Pre-covid	0.826	0.860	0.868	0.873	0.916
		Unseen	0.843	0.870	0.893	0.895	0.921

Average precision (AP) and Area under receiver operating curve (AUROC) of LightGBM and logistic regression with L2 penalty (LR-L2) for predicting 24-hour critical deteriorations on the three validation sets: ‘Complete’, the full validation set; ‘Unseen’, which includes only patients who had no admissions in the training dataset; and ‘Pre-Covid’, which includes only validation set patients admitted prior to March 1st, 2020. Each column corresponds to independent models trained with the indicated feature set concatenated to all the previous feature sets to its right.

Data-driven modelling matched or outperformed the reference model across all feature sets, with the complete feature set (rightmost group in each section of Fig. 1) giving the best performance. Both AP and AUROC trended upward as the number of predictors grew, though phenotype and supplemental observations (“& Obs”), laboratory results (“& Labs”), and clinical notes (“& Notes”) had a greater impact on the average precision while the AUROC remained more stable. Including triage and service utilisation (“& Services”) yielded the largest singular boost in AP (increase from $0.326 \rightarrow 0.513$ for LightGBM). Figure 2 illustrates the alert rate vs sensitivity and precision-recall curves for LightGBM across different feature sets and for all classifier types trained on the complete feature set. LightGBM produced fewer alerts per day on average compared to the reference model up to very high sensitivities (0.967), and all classifiers maintained an improved alert rate up to moderately high sensitivities ($>0.80$). The largest reduction of alert rate was at sensitivity 0.871, where LightGBM yielded 9.429 daily alerts, $21.165\%$ less than NEWS2’s 11.961. The positive predictive value (PPV) of LightGBM-Vitals behaves similarly to the reference model as we vary sensitivity. Performance was stable between the “Complete”, “Unseen”, and “Pre-Covid” validation sets, as shown in Supplementary Tables 4 and 5. Removing the ’known’ validation patients yielded a median increase of 0.036 for AP and 0.007 for AUROC, while validating only on admissions prior to March 1st, 2020 yielded an AP difference $<0.001$ and a median increase of 0.008 for AUROC. All models had satisfactory calibration, though with a tendency to underestimate the probability of critical deterioration, as illustrated in Supplementary Fig. 3.

**Table 3:** Summary of model performance compared to the NEWS2.

Estimator	Cutoff	Sens.	Spec.	PPV	NPV	Accuracy	F2	NNE
NEWS2	$\ge 3$	0.6021	0.8545	0.0865	0.9895	0.8489	0.2746	11.5663
	$\ge 5$	0.3968	0.9590	0.1813	0.9858	0.9465	0.3206	5.5144
	$\ge 7$	0.2201	0.9867	0.2749	0.9822	0.9696	0.2292	3.6373
LightGBM	$\ge 0.167$	0.6021	0.9735	0.3417	0.9907	0.9652	0.5225	2.9261
	$\ge 0.432$	0.3957	0.9949	0.6379	0.9863	0.9815	0.4282	1.5677
	$\ge 0.810$	0.2189	0.9996	0.9231	0.9824	0.9821	0.2583	1.0833
LR-L2	$\ge 0.114$	0.6009	0.9633	0.2726	0.9906	0.9552	0.4843	3.6679
	$\ge 0.360$	0.3968	0.9946	0.6259	0.9863	0.9812	0.4281	1.5977
	$\ge 0.788$	0.2155	0.9993	0.8832	0.9824	0.9818	0.2539	1.1323

Sensitivity, specificity, positive predictive value (PPV), negative predictive value (NPV), accuracy, F2 score, and numbers needed to evaluate (NNE) of NEWS2, GBDT (LightGBM) and logistic regression with L2 penalty (LR-L2) trained on the complete feature set. We fix the sensitivity of the models at three levels (0.602, 0.396, and 0.220) that match the observed sensitivity of NEWS2 at thresholds 3, 5, and 7, respectively.

To examine the suitability of these models for supporting track-and-trigger, we measure their performance at various cutoff points for triggering an alert. Table 3 draws comparisons with NEWS2 by fixing the models’ sensitivity at three levels (0.602, 0.396, and 0.220) that match the observed sensitivity of NEWS2 in this cohort at cutoffs 3, 5, and 7, respectively. We focus on the NEWS2 cutoff of $\ge 5$ points for triggering an emergency response, that is often adopted instead of the stricter recommended threshold of $\ge 7$ points^35^. At this operating point, LightGBM yields a PPV of 0.638, meaning we expect $63.8\%$ of patients the model deems high risk will deteriorate within 24 hours, compared to $18.13\%$ of patients occupying this NEWS2 threshold. The number needed to evaluate (NNE) for LightGBM at this sensitivity is 1.568, compared to 5.514 for NEWS2, a difference of $\sim 4$. This corresponds to requiring four fewer urgent assessments to detect one deterioration. We report the complete comparative measurements in Supplementary Table 9.

**Figure 3:**
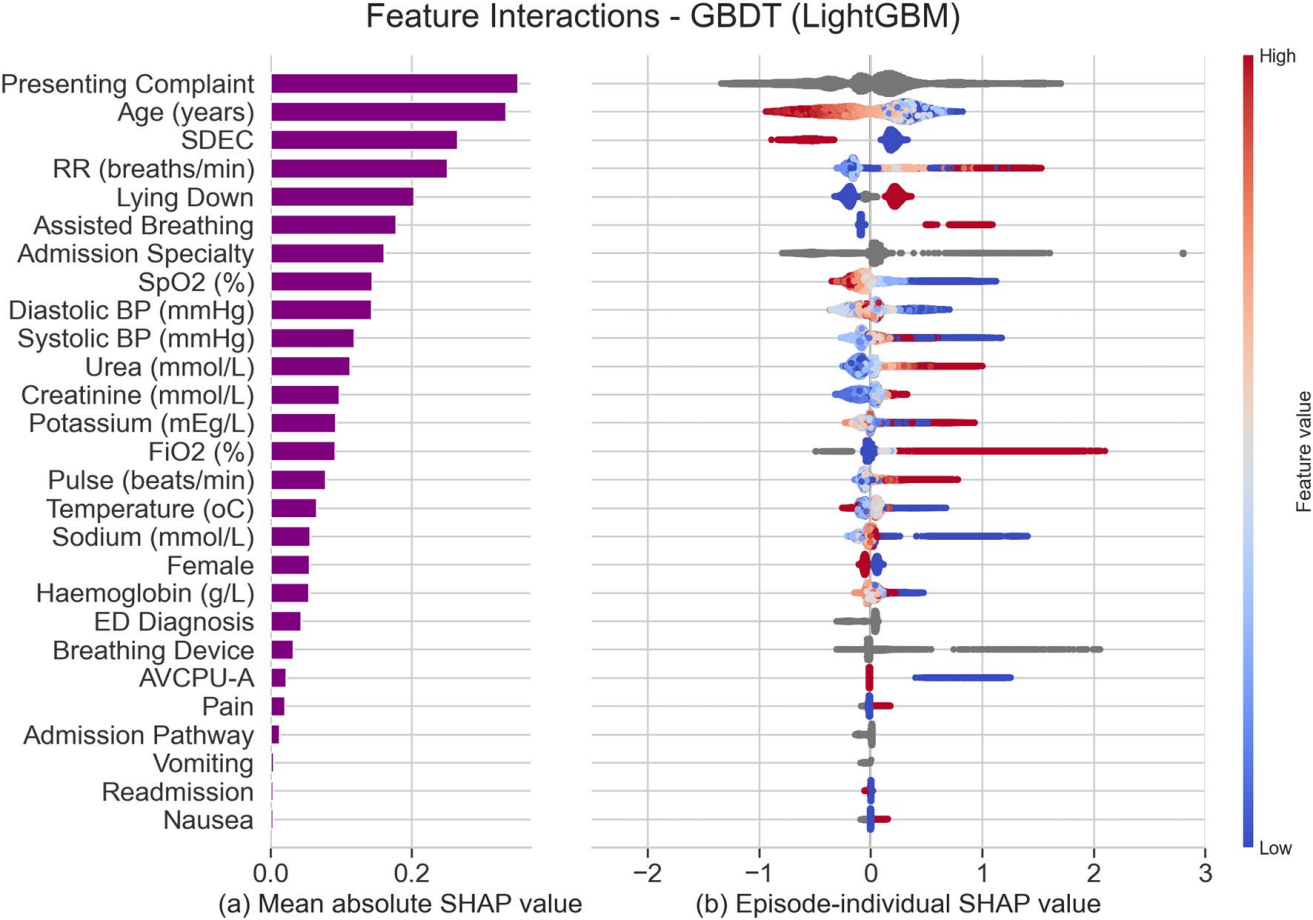
Induced feature importances for LightGBM in decreasing order of mean absolute impact. In (**a**), the bar lengths represent the mean absolute impact of each feature on the model’s predictions for the validation set. In (**b**), each point represents a value from one admission record. The points’ colour corresponds to numerical value, and their position on the x-axis represents the magnitude of their contribution towards increasing the predicted risk (if $x>0$) or reducing it (if $x<0$).

The feature interactions induced by SHAP for LightGBM allow us to compare their contribution to identifying the tracked adverse outcome. Figure 3a ranks all the included predictors by their mean absolute impact towards positive predictions (deterioration) and negative ones (no deterioration) across the validation set, (b) illustrates the patient-individual impact of each feature, and Supplementary Fig. 5 breaks down the relative impact of the values taken by categorical data features. The presenting complaint ranked the highest and contributed similarly towards positives (“diabetes”, “GI bleeding”) and negatives (“back pain”, “facial problems”). The model captured a non-linear relationship between risk and indicators of kidney function, such as creatinine and urea levels, which is consistent with clinical findings differentiating the mortality risk of acute kidney injury versus chronic disease^36^. Triage decisions were heavily influential, with same day emergency care (SDEC) invariably reducing the estimated risk, while certain clinical specialities, such as respiratory medicine, geriatric medicine, and general medicine (a catch-all for non-specialty cases), strongly contributed towards positives.

**Figure 4:**
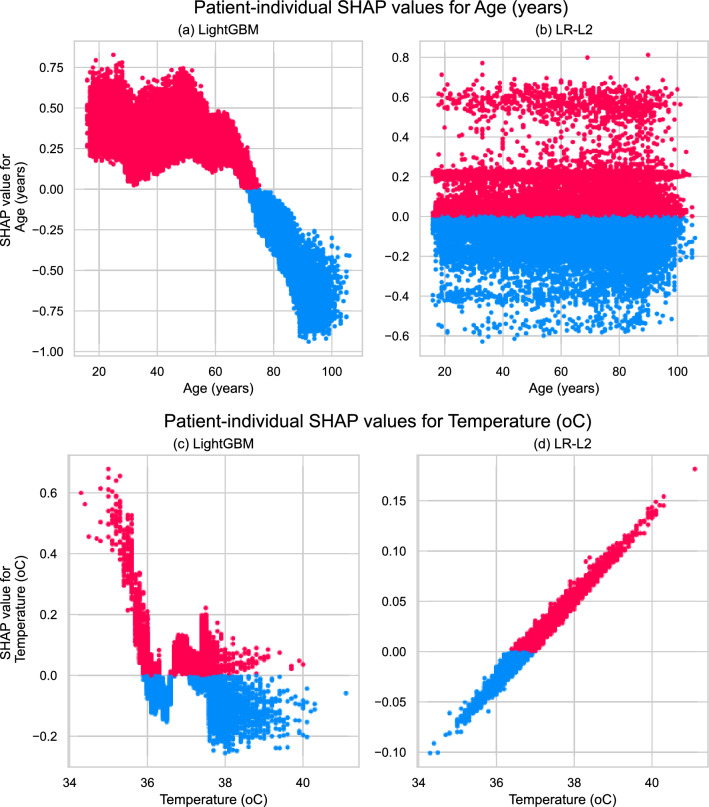
Feature-specific importances extracted by SHAP from LightGBM (**a,c**) and LR-L2 (**b,d**). The top section (**a,b**) presents the importances of patient age, while the lower section (**c,d**) presents body temperature. Each point represents a value from one validation set record. The points’ position on x-axis represents the numerical feature value, while the y-axis indicates their contribution to the prediction for that patient, with values above $y=0$ (indicated in red) contributing towards making the prediction positive and values below $y=0$ (indicated in blue) contributing towards making the prediction negative.

Similarly, we record the coefficients of the logistic regression models in Supplementary Tables 6 and 7 and find them to be consistent across the penalised models. SDEC, higher sodium levels, and specific presenting complaints (e.g. “facial problems”, “ear problems”) reduce the estimated risk. Conversely, elevated respiratory rate, potassium levels, lying down (patients flagged at the point of admission as definitely requiring a bed prior to senior review), and certain clinical specialities and breathing devices yield increased risk estimates. It is interesting to notice that age is assigned a negative coefficient. Figure 3 reveals that LightGBM also identified age as a strong predictor, with advanced age driving the model towards negative predictions rather than positive ones. We explore this non-intuitive and potentially spurious association in Fig. 4a,b which compares the two models’ patient-individual SHAP values for the age feature. We theorise this relationship is partly due to high-frailty patients (aged $\ge 80$ years), having the lowest proportion of 24-hour critical deterioration events out of all age groups (as shown in Supplementary Fig. 1) despite being very frequent attendees at the ED.

**Figure 5:**
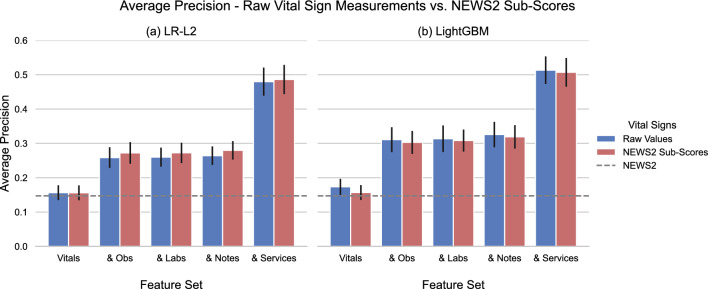
Average precision (AP) of (**a**) LR-L2 and (**b**) LightGBM. Each pair of bars corresponds to incrementally including the indicated feature sets (from Methods Table 4) as training data. For a given feature set, we measure the AP of two independently trained models, one using the direct measurements of vital signs (blue), and one with the vital signs encoded using the NEWS2 severity scales (red). The error bars represent $95\%$ bootstrapped confidence intervals.

As an additional test, we trained logistic regression and LightGBM models with vital signs encoded into integers $0-3$ per the NEWS2 severity scales^6^. We compared the results with the classifiers’ performance when using the original vital sign values to investigate how each model type captures the non-linear relationship between vitals and clinical outcomes in Fig. 5. We observe that the ’handcrafted’ scales boosted the performance of logistic regression across feature sets, while LightGBM’s performance either dropped or remained stable. Figure 4c,d presents an example of a diverging relationship learned by LightGBM and LR from the same feature, temperature. Note that the presented results thus far assume 24 hours after admission as the cut-off point for identifying deterioration events. Supplementary Fig. 4 illustrates how the AUROC of LightGBM and LR-L2 varied when we increased the (cumulative) time threshold gradually from 24 hours to 30 days. Across all feature sets, the AUROC peaked at the first 24–48 hours and then trended downwards as the cut-off widened and the on-admission measurements for each newly included sample became more distant from the outcome.

**Figure 6:**
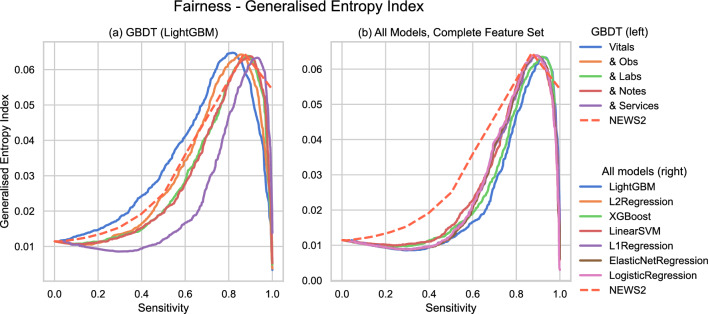
Generalised Entropy vs Sensitivity curves of (**a**): LightGBM across the tested feature sets, and (**b**): All classifier types trained on the complete feature set. We plot each model’s generalised entropy index for a given sensitivity value (y-axis) against that sensitivity value (x-axis). A lower value on the y-axis indicates a more fair distribution of ’benefit’, i.e. of receiving a positive prediction. A theoretical ’perfect’ model would yield a single point (0, 1) in the lower-right corner of the plot.

Finally, Fig. 6 presents the generalised entropy index vs sensitivity for LightGBM across the tested feature sets and all models trained on the complete feature set. Supplementary Fig. 6 isolates the between-group fairness component of the generalised entropy index when we consider the population groups defined by the protected demographic characteristics of age group and sex (as specified in Supplementary Fig. 1). All models except for LightGBM-Vitals achieve an improved fairness score compared to the reference model across sensitivity thresholds. NEWS2 produces a better between-group fairness and, correspondingly, a more significant unfairness within the demographic groups, under the complete feature set above sensitivities of $\sim 0.85$. To account for potential pre-existing inequalities in the cohort, we record the differential bias amplification of the models in Supplementary Table 8. These measurements corroborate the generalised entropy findings, with a positive bias amplification under the vital signs feature set when considering age groups. However, this diminishes when considering intersectional protected groups of both age and sex. Bias amplification values across all other feature sets are strongly negative - indicating removal of bias - or near zero. We theorise that this unusual amplification of inequality with respect to age is due to the vital signs feature set containing insufficient information to predict our tracked outcome correctly for patients of all ages.

## Discussion

In a large cohort of ED admissions, we developed and validated predictive models that can differentiate patients likely to deteriorate shortly after admission. GBDT methods received the most focus as they are state-of-the-art for sparse classification tasks (even compared to deep neural networks^37^), they can capture non-linear interactions such as those present in clinical data, and they natively incorporate missing values, which are inevitable under typical clinical workflows. Using our trained models’ coefficients and the extracted global justifications, we can identify which characteristics of our cohort were most predictive of the tracked clinical outcome both on the patient level and across the studied population. Features that encode the clinical context of the patient’s condition, presentation, and comorbidities stood out as the most useful. These included presenting complaints, triage decisions such as the utilisation of SDEC, and the assigned clinical speciality, among others. Patient age stood out for being inversely correlated with our tracked outcome, against clinical intuition^38^, which we theorise results from the low prevalence of the outcome within the highest age band. While it did not result in the model amplifying unfair bias, it presents a clear example of model interpretability revealing spurious associations that might require correcting prior to deployment.

Our cohort of patients with varied acuity and conditions reflects a typical real world ED acute medical workload. Frontline staff collected the patient data under everyday conditions, where operational pressures affect the timeliness and reliability of data entry. We excluded little data since, although comprehensive manual data curation is helpful for model development, it conflicts with scalable deployment and real-time use of data-driven systems^27^ and can lead us to discard valuable information for uncommon cases^39^. We did not carry out a priori feature selection but instead used all available data and employed modelling methods that perform intrinsic feature selection and can differentiate useful features based on evidence. Healthcare digitalisation is an ongoing process^40^, so we made no assumptions about the level of EPR integration. Instead, through our experiments with different feature sets, we accommodate different levels of data availability. The lack of a standardised benchmark dataset makes direct comparisons between studies on this topic challenging, so we minimised centre-specific assumptions and standardised our modelling pipelines’ structure to establish reproducibility.

We similarly designed our assessment methodology around the extant practical challenges and presented results with the context of their resource cost. We used a temporal split of the study data to assess performance but retained the records where the patient had presented to the same ED during the training period as frequent repeat attendees reflect the reality of clinical practice. To strengthen our results, however, we also examined removing these records and still demonstrated good performance. Calibration is often underappreciated^10^, and alert frequency deserves attention as alert fatigue is a key critique aimed at existing solutions from frontline staff^21^. We focused on measuring discriminative skill and avoided setting a threshold for positive or negative classifications, as setting it carries clinical, operational, and ethical complications. Directing care where it is needed promptly is vital and far outweighs the cost of false positives. However, excessive false alarms are detrimental to a model’s utility due to alert fatigue^41–43^. Balancing clinical risk against available capacity is a well-researched problem beyond the scope of our study^23,44^; instead, we argue that early-stage researchers should aim to maximise the discriminative skill of their model, as might be measured by AUROC or the highest achievable sensitivity while preserving acceptable specificity.

Our predictive models have the potential to positively impact clinical practice. Track-and-trigger systems’ intended purpose is to identify patients at imminent risk of deterioration, leading to mortality, admission to critical care, or cardiac arrest^9^. However, limited resources lead to a conflict when trying to direct care to the right patient at the right time^45^; the nominal NEWS2 trigger threshold of 7 does not capture the majority of patients at imminent risk of an adverse outcome, while physiological decline has been found to commence at a NEWS2 threshold of 3^46^. Consequently, studies have aimed to augment the standard NEWS2 with additional predictors, such as biomarkers^47,48^. Modified EWS are typically compared with the NEWS2 via their AUROC. Although they often show statistical improvements in performance, their practical and clinical benefit remains open to debate. Our proposed model includes multiple and varied predictors, providing a more comprehensive patient assessment. In a deployment setting, if the decision threshold for identifying high-risk patients is set to match the NEWS2, our models would flag fewer cases, reducing the resources needed to maintain the same level of care. If the decision cutoff is softened to match the NEWS2’s observed alert rate, the sensitivity increases, allowing our models to identify cases currently missed by the NEWS2. If incorporated into EPR, they could provide clinicians with automatic alerts, flagging high-risk patients for urgent clinical review and highlighting the patients’ characteristics that led to that assessment. The next developmental step is to trial our models in clinical practice and assess their real-world performance, practical feasibility, and acceptability to clinicians.

Our observational dataset is limited to one acute secondary care centre, but many measured parameters and outcomes vary between providers. Even near-universal predictors such as vital signs may be measured differently. For example, manual measurement of respiratory rate is less precise than an electronic recording^49^, provision of supplemental oxygen is subjective and depends on operational constraints, availability, guidelines, and expertise^50^, and the same oxygen saturation may represent different levels of clinical risk depending on whether it was measured before or after commencing oxygen^51^. Furthermore, we recorded symptoms, vital signs, and laboratory results from the point of admission. This information gives a cross-sectional view of the patient’s condition as seen by the admitting clinician but excludes longitudinal information, which prior work has collected via continuous vital sign monitoring and used to train highly effective models^33,52^. Finally, we investigated unfair bias and group inequalities in the models to the best of our ability but limited our assessment to the available protected characteristics. While patients face divergent clinical risks depending on characteristics such as sex, age, or ethnic background^53^, finer data such as economic stability, education, community context, and other social determinants of health are also strong predictors of clinical risk^54^. We recommend that researchers investigate fairness thoroughly, especially if the models they construct are intended to autonomously screen or prioritise patients’ access to care, to ensure healthcare inequalities are not perpetuated^55^.

There are key considerations researchers should take into account before adopting similar modelling methodologies. It is essential to consider the validity of jointly modelling outcomes and the reliability of any composite outcome as a surrogate for clinical deterioration. We considered critical care admission and mortality as a single outcome because we expect both to be preceded by deranged physiology, and the clinical response to both, in terms of urgency and skill, is similar^8^. The joint outcome served as a surrogate for any severe and time-sensitive medical condition encountered at the ED; this is a common modelling choice in the literature^10,56^ and one we find reasonable, as our focus is on clinical escalation, which is the primary purpose of an EWS^9^. However, critical care and mortality represent competing outcomes as the former intends to prevent the latter^57^. Future studies may prefer to avoid such assumptions and investigate multiclass modelling or compositing multiple binary classifiers, each trained to identify a single measurable outcome. Some features we utilised, such as triage outcomes, directly represent clinical decision-making. Their inclusion is in contrast with the ’one-size-fits-all’ approach taken by the NEWS2^58^ or their explicit exclusion by some studies to avoid capturing and amplifying human-originated bias^27^. If the purpose of a system is to ’sense-check’ clinical decisions, its input data should ideally be as isolated as possible from those decisions. However, our findings show that these features efficiently stratify patient risk, making them valuable for producing reliable clinical risk estimates as long as the risks are made clear and considered.

In conclusion, we demonstrated the development of predictive models on a large, real-world sample of general ED patients. Considering the high and rising pressures EDs face and the potential for missed diagnoses, models built from continuing our work could be clinically valuable for decision support. We contend that this study demonstrates the power of machine learning for modelling or adapting to patient populations for this task. By incorporating modularised modelling pipelines from contemporary machine learning practice and leveraging the advances in interpretable modelling, we encourage future research to follow a systematised model-building approach and help obtain clinically useful prognostic tools.

## Methods

### Data collection and preparation

#### Methodology

 This is a retrospective observational study of routinely collected patient-level data. As this study concerns the development of a predictive model, we follow the guidance set out in the Transparent Reporting of a Multivariable Prediction Model for Individual Prognosis or Diagnosis (TRIPOD) statement^59^. We provide the TRIPOD model development checklist in Supplementary Table 10.

#### Study setting

 Salford Royal Hospital is a digitally mature, ’paper light’ NHS secondary care hospital with over 100, 000 Emergency Department attendances and $\sim 40,000$ unplanned admissions annually. The Hospital’s EPR captures clinical episode data in real-time from arrival at the ED until discharge. Selected data are exported pseudonymously to an internal data warehouse to drive local quality improvement and service development projects. Our study considered all such records from 1st January 2015 to 31st March 2022. This starting date reflects the first calendar year after the introduction of electronic NEWS recording in the Hospital. We selected all patients aged $\ge 18$ years admitted to the Emergency Admissions Unit (EAU) that had a sufficiently long stay for their first NEWS/NEWS2 to be recorded. The EAU predominantly treats patients admitted to a conventional Acute Medical Unit (AMU) but also accepts patients from all specialties. Our data include patients who received ambulatory emergency care (AEC) and same-day emergency care (SDEC^60^) but exclude planned admissions, day case reviews, and maternity cases. We further exclude a small subset of patients that received critical care interventions at the ward on-arrival, such as invasive ventilation or cardiac pulmonary resuscitation, without being moved to critical care or admitted under critical care medicine. Supplementary Fig. 2 summarises our exclusion criteria and subsequent data splitting. We present summary statistics of the dataset in Table 1, and further details of the collected categorical features in Supplementary Table 3.

#### Data collection

 Most acute admissions arrive to the ED, while a smaller minority are admitted directly to the EAU or via ambulatory emergency care. Therefore, initial observations and investigations are taken at the point of entry to hospital. As a routine part of EAU admission, the responsible staff member (nurse or support worker) records the patient’s vital signs within a target of 30 minutes of arrival. The vital signs that make up the NEWS2^6^ are measured in a standardised manner using Dinamap monitors, and manually transcribed into EPR. These data are body temperature ($^{\circ }C$), heart rate (beats/min), systolic (and diastolic) blood pressure (mmHg), and peripheral oxygen saturation ($\%$). Other parameters are measured using manual observation and direct questions. These are the patient’s level of consciousness (AVCPU), presence of pain, nausea, or vomiting, whether the patient was receiving oxygen at the time of SpO2 measurement and, if applicable, the oxygen flow rate and mode of delivery.

Independent of this, blood test results are automatically recorded in the laboratory information management system (LIMS) and copied to EPR in real time. Whether a patient receives routine blood tests depends on operational pressures and considerations at the ED, not on the patient’s presentation. Other information available upon arrival at the EAU includes identifier data such as the unique patient number; basic phenotypic information, such as their age and sex; admission pathway (e.g. ED, emergency GP referral); arrival time; and unstructured notes indicating their presenting complaint and the ED staff’s primary diagnosis. For patients with prior hospital visits, significant comorbidities and previous admission events are available from the point of admission.

Following initial collection, our data are supplemented with downstream administrative and outcome information. Final admission diagnoses and treatment are measured using ICD-10-CM, OPCS-4, and HRG codes, alongside service utilisation records. The ICD-10-CM diagnoses are compiled after discharge by a clinical coding team, drawing form information recorded in the EPR. Procedures and service utilisation are similarly recorded in EPR and coded retrospectively using OPCS-4. We do not use the retrospectively coded diagnoses or procedures as model training inputs, but instead for data filtering or delineating subpopulations in the cohort for more detailed model evaluation. Each ward transfer and length of stay (LOS) per ward are provided in chronological order. Outcome parameters include inpatient and post-discharge community mortality, 30-day readmission, date and time of discharge, and total LOS.

#### Ethical approval

 All data used in this study is collected as part of routine clinical care. In keeping with Health Research Authority guidance, an application to the Integrated Research Application System (IRAS) and Confidentiality Advisory Group (CAG) approval were not required as the data controller (The Northern Care Alliance NHS Foundation Trust) deemed that the use of non-identifiable and anonymised patient level data did not contravene a breath of confidentiality. Local approval to undertake the study was granted by the Trust’s Research and Innovation Department (R &I internal reference 21HIP13). All methods were carried out in accordance with relevant guidelines and regulations.

#### Feature engineering

 Some of the collected data is not directly clinically relevant or may be unsuitable for modelling under a realistic use case. However, we can use it to engineer useful features. Other features are relevant but first require cleaning or modification. We derive the following features:

- 30-day readmission. We mark as readmissions those patient records that are preceded by a record bearing the same unique patient ID if the two records’ admission dates are $\le$ 30 days apart.
- Unstructured clinical (ED) notes. The presenting complaint and ED diagnosis are unstructured text and thus could hold any string value. We cluster presenting complaints into a categorical variable representation since the 50 most frequent values account for nearly all records ($97.58\%$), and we assign the remainder a sentinel value. In contrast, the ED diagnosis varies greatly between records, so we compile a list of clinically relevant word stems and abbreviations based on expert opinion and construct a boolean Bag-of-Words vector for each record indicating which ones are present. We provide the prevalent presenting complaint values and diagnosis stems in Supplementary Table 3.
- Vital signs. We investigate training models directly on vital sign readings or encoding them into integers $0-3$ per the NEWS2 severity scales^6^. The former approach forces models to form evidence-based weightings for values that correlate with adverse patient outcomes, while the latter allows us to incorporate the domain knowledge embedded in the NEWS2 into the models. Recorded vitals must be checked for spurious values as they are the only parameters transcribed into EPR manually under a typical workflow. We check each record against fixed ranges (e.g. 0–100$\%$ for SpO2) and soft thresholds based on the range of physiologically possible values determined by expert clinical opinion. We provide further details on filtering these values in Supplementary Table 1.

#### Data labelling

 Our tracked outcome is a composite of in-hospital mortality or admission to critical care from the ward within a specified time threshold after presenting to the ED. The criteria to identify patient episodes that belong in the positive class are are:

- The discharge/end-of-episode record indicates the patient died in the hospital AND the record’s timestamp is within 24 hours of the admission timestamp, OR.
- Their service utilisation indicates admission to critical care or provision of critical interventions on the ward AND this occurred within 24 hours of the admission timestamp.

We identify critical care based on recorded admission into the hospital’s critical care unit (CCU) or the high-dependency medical unit (H1). We use the length-of-stay per ward to determine how long after the patient’s arrival they were admitted to critical care. A smaller subset of patients received critical care interventions without being moved to these wards, and we can detect most such cases through specific entries in their recorded procedures - OPCS-4 codes E85.1 (invasive ventilation), X50.3 (advanced cardiac pulmonary resuscitation), X50.4 (external ventricular defibrillation), or X56.* (intubation of the trachea).

### Model development

#### Modelling pipeline

 We adopt a modularised model-building approach from contemporary machine learning practice. We consider *pipelines* as sequences of distinct tasks in the model-building process, where each task’s output becomes the subsequent task’s input. Some tasks modify the data samples in preparation for modelling. At least one task in each pipeline is a learning/model-building algorithm. Then, subsequent post-processing tasks may alter the predictive model’s output or aggregate multiple models. We implement the following tasks, executed in order:

1. Data pre-processing. Executes the data preparation tasks outlined previously to produce a vector representing each patient episode. We parameterise the processing component to include only the features we specify, so we may investigate selectively including features and the impact they have on performance. The sets of features we consider are listed in Table 4.
2. Data splitting. Partitions the data into two subsets; we use one for model construction and reserve the second for validation. We prefer a temporal train-test split over standard random splitting^61^, and partition the dataset such that the first 2/3 of records chronologically serve as the training set and the latter 1/3 as the validation set. For some experiments we implement an additional filter that excludes any validation set records where the patient, as identified by their unique ID, had also appeared in the training set in a previous admission.
3. Data imputation. Supplements standard values into data samples with empty fields. We apply this only to those modelling algorithms that are incompatible with missing data in their inputs (logistic regression). We impute numerical features with the median over the training dataset and binary and categorical variables with appropriate constant values. The imputed values correspond to a patient in stable condition.
4. Model construction. A learning algorithm receives the data samples and produces a predictive model.
5. Calibration. As a post-processing step, we map the numerical outputs of the trained predictive model into well-calibrated probabilities, substituting the model’s original output $C(\varvec{x_i})$ on input $\varvec{x_i}$ for an estimate of $Pr(y_i=1|C(\varvec{x_i}))$, the conditional probability of belonging to class $y_i$. We opt for isotonic calibration^62^ and fit a meta-estimator that learns the isotonic (monotonically increasing) mapping *m* that minimises a loss function $\mathcal {L} = \sum _i w_i(y_i - m(C(\varvec{x_i}))^2$.

**Table 4:** Dataset features and units categorised into feature sets.

Feature set	Features (units)
Vital signs (NEWS2)	Body temperature ($^{\circ }C$), heart rate (beats/min), systolic blood pressure (mmHg), peripheral oxygen saturation ($\%$)
Supplemental obs. & phenotype	Sex (M/F), Age (years), Diastolic blood pressure (mmHg), breathing device (if applicable), prescribed oxygen (FiO_2_), presence of pain (Y/N), presence of nausea (Y/N), presence of vomiting (Y/N), lying down* (Y/N)
Clinical notes	Presenting complaint (text), ED diagnosis (text)
Laboratory results	Haemoglobin (g/L), urea (serum, mmol/L), sodium (serum, mmol/L), potassium (serum, mEq/L), creatinine (mcmol/L)
Service utilisation	Triaged to SDEC (Y/N), readmission within 30 days (Y/N), admission speciality (category), admission pathway (category)

In the given units, “Y/N” indicates binary variables, “category” un-ordered categorical variables, “text” unstructured text data, and “M/F” indicates male or female.

#### Model training and tuning

 We construct pipelines with each combination of available components. For each one, we execute a single-objective Bayesian optimisation process (Tree-Structured Parzen approach^63^) to sweep over the space of possible hyperparameter values and probabilistically settle on values that maximise our chosen performance metric, average precision. We construct the final models using the best-scoring hyperparameters after 1000 tuning iterations. We report the resultant hyperparameters in Supplementary Table 2. We avoid training the calibration meta-estimator on the same data that trained the classifier and, instead, we combine calibration with k-fold cross-validation. We randomly separate the training dataset into *k* equal-sized partitions (setting parameter $k=5$), train a model on four of the subsets and fit the calibrator using the remaining subset. We iteratively repeat this *k* times to such that each partition serves as the calibration set once and produce *k* independent models to serve as sub-estimators of a model ensemble. The final ’representative’ probability prediction of the ensemble *C* of sub-estimators $C_1, \cdots , C_k$ for input vector $\varvec{x}$ is taken to be the arithmetic mean of the sub-estimators’ predictions: $C(\varvec{x}) = \frac{1}{k} \sum ^k_{i=1} C_{i}(\varvec{x})$.

#### Model evaluation

 We assess the discriminative skill of the models by constructing the precision-recall curve and measuring the average precision, which is the mean of the PPV (or precision) over the interval of sensitivity (TPR/recall) values from 0-1. We approximate this with the weighted mean of the measured PPV across the observed sensitivity thresholds, where the weight of each element is the difference in sensitivity from the previous element^64^.

$$\begin{aligned} AP = \int _0^1 p(r)dr \approx \sum ^n_{k=1} P(k)\Delta r(k), \end{aligned}$$

where *p*(*r*) is the PPV as a function of sensitivity *r*, *P*(*k*) is the precision at cut-off *k* in the ranked sequence of data samples in the validation dataset, and $\Delta r(k)$ is the difference in recall $r_k - r_{k-1}$. We calculate the confidence intervals for our estimate of the AP by bootstrapping with 1000 bootstrap samples over the validation set^65^. We construct the PR curve by plotting the PPV on the y-axis against sensitivity on the x-axis^66^. On the PR curve, an unskilled model giving random outputs would yield a horizontal line at $y=P/(P+N)$, where *P* and *N* are the numbers of positive and negative samples in the data, respectively, while a theoretical ’perfect’ model would yield a single point (1, 1) in the upper-right corner of the plot.

We construct the receiver-operating characteristics (ROC) curve and compute the area under the receiver operating curve (AUROC). We plot the false-positive rate (1 minus the specificity) on the x-axis against the sensitivity on the y-axis. The minimum possible area under the curve is 0.5, corresponding to a completely random relationship between the model’s output and the ground truth. Generally, $0.7-0.8$ indicates reasonable discrimination, and values over 0.8 indicate good discrimination^8^. We compute confidence intervals for the AUROC as before.

The ROC and PR curves both provide a model-wide evaluation and, while the ROC curve is more common, we prefer the PR curve because it better indicates the skill of the model at predicting the minority (positive) class correctly and is less influenced by predicting the majority (negative) class correctly^67^. The PR curve further allows us to visually inspect how quickly PPV deteriorates as we increase model sensitivity^66^, which is helpful in a task where it may be appropriate to value sensitivity over specificity.

Finally, we investigate how a model’s daily alert rate varies with sensitivity^16^. We construct an alert rate curve by plotting the alert rate (the number of positive predictions divided by the number of days) on the y-axis over sensitivity on the x-axis. The point where two lines intersect corresponds to the maximum achievable sensitivity for which the model with the lower line maintains a lower daily alert rate than the model with the upper line.

#### Model bias

 We investigate two forms of undesirable bias: individual, representing how dissimilarly we treat individuals who deserve similar outcomes^68^, and group-based, measuring the inequality of predictions between demographic groups defined by protected characteristics^69^. The generalised entropy index^70^ applies to both notions concurrently. Given a patient record $\varvec{x}_i$ with ground-truth outcome $y_i$, we define the *benefit* experienced by the patient due to model prediction $C(\varvec{x}_i)$ as:

$$\begin{aligned} b_i = y_i - C(\varvec{x}_i) +1, \end{aligned}$$

Under this representation, a false-positive patient experiences a large benefit ($b=2$), while a false-negative that the model missed has the heaviest penalty ($b=0$). Then, given the vector of benefit values over the validation set, $\varvec{b} = (b_1, b_2, \cdots , b_n)$, and their arithmetic mean $\mu (\varvec{b})$, we measure the generalised entropy index fairness score $\mathscr {E}^2_{\varvec{b}}$, where:

$$\begin{aligned} \mathscr {E}^{\alpha }(\varvec{b}) = \frac{1}{n \alpha (\alpha -1)} \sum _{i=1}^n \left( \left( \frac{b_i}{\mu (\varvec{b})} \right) ^{\alpha }-1 \right), \end{aligned}$$

Furthermore, given protected groups $g \in G$, with each comprising $n_g$ patient records with benefit vectors $\varvec{b}^g = (b^g_1, b^g_2, \cdots , b^g_{n_g})$, we decompose the generalised entropy into its between-group component $\mathscr {E}^2_{\beta }$ and its within-group component $\mathscr {E}^2_{\omega }$, representing group and individual fairness, respectively. We measure the between-group component $\mathscr {E}^2_{\beta }$, where:

$$\begin{aligned} \mathscr {E}^{\alpha }_{\beta }(\varvec{b}) = \mathscr {E}^{\alpha }(\varvec{b}) - \mathscr {E}^{\alpha }_{\omega }(\varvec{b}) = \sum ^{{|}G{|}}_{g=1} \frac{n_g}{n \alpha (\alpha -1)} \left( \left( \frac{\mu (\varvec{b}_g)}{\mu (\varvec{b})} \right) ^{\alpha }-1 \right), \end{aligned}$$

We define demographic groups based on the available protected characteristics - age and biological sex. We partition the continuous age variable into age groups, as illustrated in Supplementary Fig. 1. For both scores, the ideal value is 0 and higher values indicate unfair classification.

We additionally compute the differential fairness bias amplification exhibited by our models^71^. The differential fairness metric is defined from the standpoint of intersectionality, i.e. equally protecting population sub-groups defined by multiple overlapping protected characteristics. Bias amplification measures a predictive model’s unfairness compared to any pre-existing bias reflected in the dataset due to inequality in the real-life generative process of the data. Given a set of patient records $\varvec{x}$ and protected groups $(g_i, g_j) \in G \times G$, the (smoothed) differential fairness $\varepsilon$ of a classifier *C* is defined by the relation:

$$\begin{aligned} e^{-\varepsilon } \le \frac{\sum _{\varvec{x} \in g_i} C(\varvec{x}) + \alpha }{{|}g_i{|} + {|}R_Y{|}\alpha } \frac{{|}g_j{|} + {|}R_Y{|}\alpha }{\sum _{\varvec{x} \in g_j} C(\varvec{x}) + \alpha } \le e^{\varepsilon }, \end{aligned}$$

where ${|}R_Y{|} \alpha$ is the Dirichlet smoothing concentration parameter (we set $\alpha = 1.0$, assuming no prior information). Then, the bias amplification metric is defined as the difference $\varepsilon _{C} - \varepsilon _{D}$ of the differential fairness value for the model *C* minus the value for the dataset *D*’s ground truth. A negative bias amplification indicates that the predictive model reduces differential unfairness, while a positive value means the estimator is more biased than the original data.

### Supplementary Information

Supplementary Information.

## Data Availability

The datasets generated and/or analysed during the current study are not publicly available due to the data sharing agreement between the Northern Care Alliance NHS Trust and Durham University, but are available from the corresponding author on reasonable request.
