# Supplementary material for: Predicting acute clinical deterioration with interpretable machine learning to support emergency care decision making
Source: Sci Rep. 2023 Aug 21;13:13563. doi: 10.1038/s41598-023-40661-0 (PMC10442440; doi:10.1038/s41598-023-40661-0)
Supplement: Supplementary file 1 — Supplementary Information. [file 41598_2023_40661_MOESM1_ESM.pdf]

Predicting Acute Clinical Deterioration with Interpretable Machine Learning to support Emergency Care Decision Making

Supplementary Material

Data Preparation

We filter all manually recorded data features using the ranges in Table 1. Where a value is missing or falls outside these ranges, we replace it with a value inferred from the recorded NEWS2 sub-score. Specifically, we set it to be the midpoint of the relevant NEWS2 range. Where the sub-score has not been recorded but the raw value is present (and not invalid), we use the value to compute the NEWS2 sub-score ourselves.

Table 1. Valid ranges for manually recorded data features.

Variable	Range	Unit
SpO2	$40-100$	%
Systolic BP	$40-300$	mmHg
Diastolic BP	$20-200$	mmHg
Temperature	$25-45$	°C
Pulse	$25-300$	Beats/min
Respiration Rate	$5-80$	Breaths/min

Furthermore, we apply the following feature-specific filtering:

- **O2 Saturation (SpO2).** The NEWS2 specification gives two scales for this parameter:
  - $SpO2_1$: By default.
  - $SpO2_2$: For patients with a prescribed oxygen saturation requirement of $88-92\%$ (e.g., in patients with hypercapnic respiratory failure).

  Since the choice of scale is determined by the responsible clinical staff on a case-by-case basis, we use the following criteria to infer which scale to use when re-computing the NEWS2 sub-score for SpO2:

  - Patients receiving oxygen using NIV, as recorded directly or in their set of coded procedures (OPCS-4 E85.2).
  - Patients with COPD (as determined by ICD-10 coded diagnosis J44.*) AND receiving oxygen via Venturi 24 or 28.
  - Patients with COPD (as above) and SpO2 $< 88\%$.

  Finally, if the patient is receiving supplemental oxygen, there is ambiguity as to whether a high NEWS2 oxygen sub-score indicates very high or very low saturation. In that case, we mark the value as missing. Patient records pre-dating the introduction of NEWS2 use the original NEWS scale ($SpO2_1$) for their O2 saturation score.

- **Respiration Rate.** In addition to the range given in Table 1, we assume triple-digit values to be erroneous entries of two-digit values (e.g., $250 \rightarrow 25.0$).

- **Oxygen Flow Rate.** This supplemental parameter is recorded in mixed units (Litres/min or FiO2). We translate all values to FiO2 where possible:
  - Values $1-15$ are inferred to be in Litres/min.
  - Decimal values are inferred to be FiO2, with the exception of 0.5.
  - Values of 0.5 and any remaining values are determined based on the device used to deliver the oxygen. Nasal cannula and simple mask correspond to Litres/min, while other devices correspond to FiO2.

  We convert Litres/min to FiO2 using the formula $FiO2 = 0.2 + (Litres/min * 4)/100$.

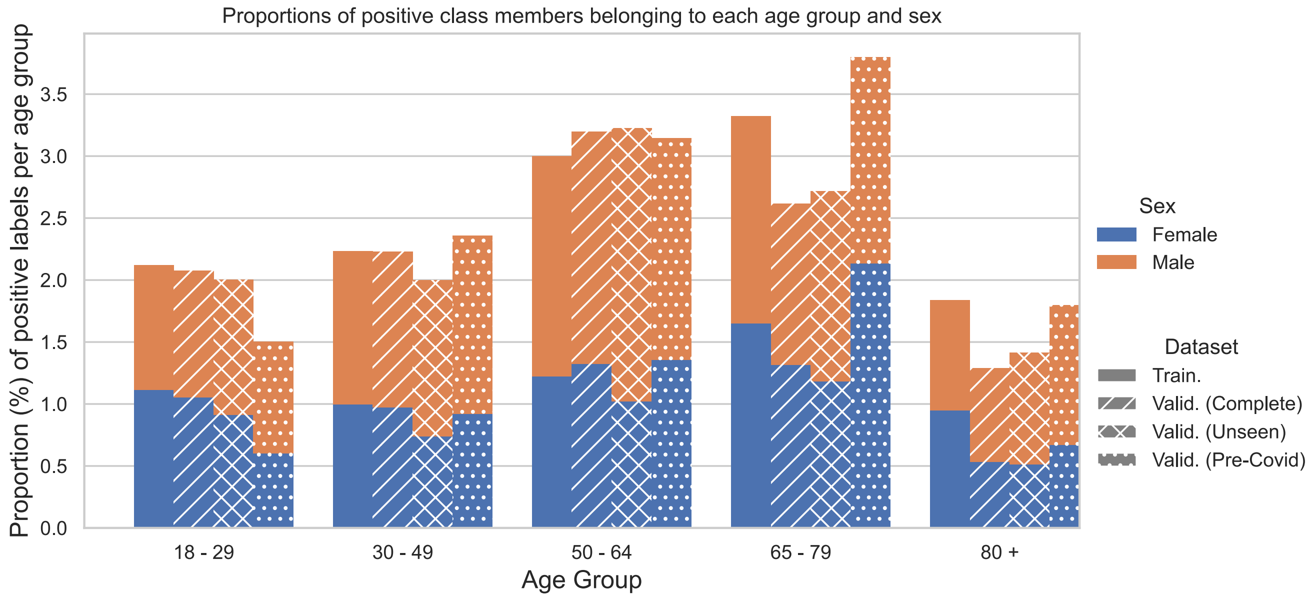

Figure 1. Outcome distribution across age groups and biological sex. Each bar length represents the proportion of positive labels (identified deterioration outcomes) of the corresponding dataset that belonged to the indicated demographic group.

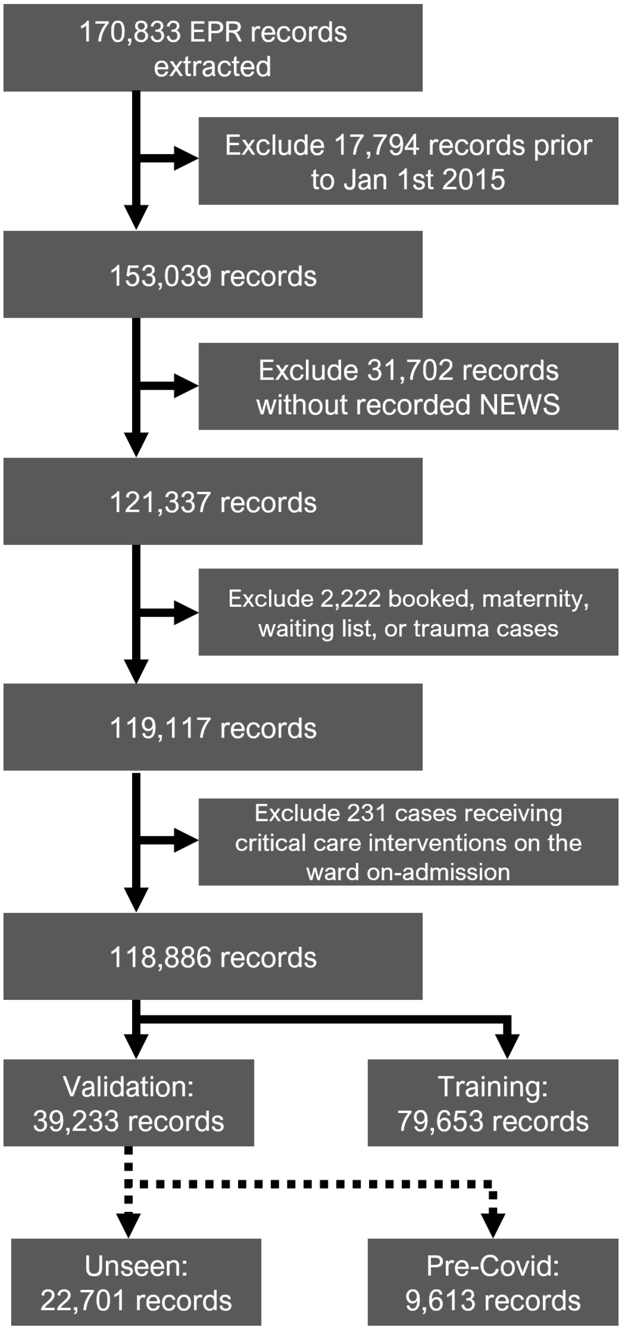

Figure 2. Flow diagram indicating the inclusion and exclusion of patient records extracted from EPR in the study.

Implementation

Table 2. Hyperparameters for each model across the examined feature sets. These values were chosen using bayesian optimisation after 1000 iterations. Any omitted parameters were assigned their default values.

Model	Parameter	Vitals	& Obs	& Labs	& Notes	& Services
**LR**	class_weight	None	None	None	None	None
**LR-EN**	C	0.0592	8.4126	1.1313	8.2228	7.0032
	class_weight	None	None	None	None	None
	l1_ratio	0.4133	0.355	0.9356	0.5862	0.8086
**LR-L1**	C	4.1169	2.8061	7.6951	4.4909	0.2121
	class_weight	None	None	balanced	balanced	None
**LR-L2**	C	5.7762	8.6765	9.9825	9.9151	3.5386
	class_weight	None	None	None	None	None
**LightGBM**	colsample_bytree	0.6075	0.989	0.5526	0.4737	0.4804
	is_unbalance	True	False	False	False	False
	min_child_samples	120	147	73	23	147
	num_leaves	29	63	16	202	13
	reg_alpha	2.6785	0.0402	1.5138	8.8073	0.0001
	reg_lambda	9.9052	0.0009	0.0941	0.0024	9.5277
	scale_pos_weight	None	53	2	4	2
	subsample	0.8714	0.958	0.5702	0.9042	0.4902
	subsample_freq	4	4	1	2	1
**LinearSVM**	alpha	0.0032	0.0004	0.0002	0.0011	0.0005
	class_weight	balanced	None	balanced	balanced	balanced
**XGBoost**	alpha	0.0	0.0	0.0	0.0	0.0
	colsample_bytree	0.8389	0.6157	0.6273	0.4414	0.597
	eta	0.0196	0.0454	0.0231	0.00004	0.2193
	gamma	0.0	0.0	0.0005	0.0	0.0
	grow_policy	lossguide	lossguide	lossguide	depthwise	lossguide
	lambda	0.0372	0.0019	0.0624	0.157	0.3922
	max_depth	9	7	9	9	3
	min_child_weight	8	9	6	5	4
	scale_pos_weight	3	6	6	15	81
	subsample	0.2505	0.7236	0.4115	0.3563	0.9937
	tree_method	approx	hist	approx	approx	approx

Results

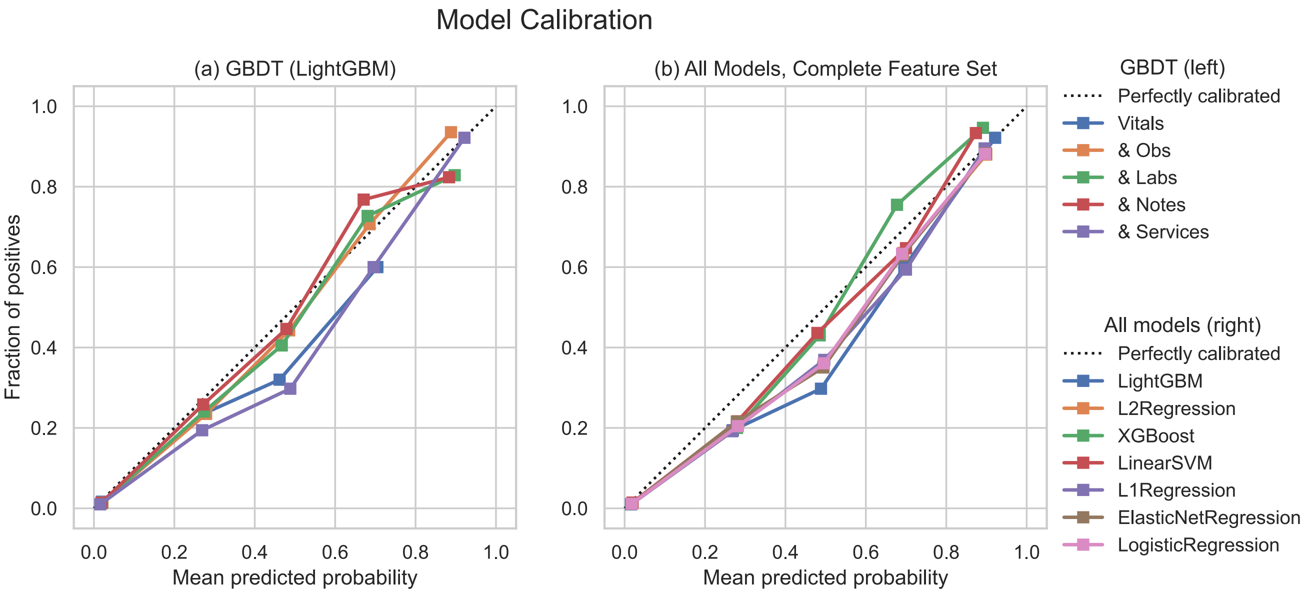

Figure 3. Calibration Curves for **(a)**: LightGBM across feature groups (concatenated incrementally) and **(b)**: All learning algorithms trained the complete feature set (equiv. "& Services"). Curves are plotted from each model's prediction outputs for the complete validation set.

Table 3. Summary of categorical features in the study sample. Each table section (except for ED Diagnosis) presents the most prevalent values for each feature, with the remaining values represented by "Other".

Variable	Value	Total	Train.	Valid. (Complete)	Valid. (Unseen)	Valid. (Pre-Covid)
**Admission Pathway**	**Emg. A&E**	110627 (93.05%)	74286 (93.26%)	36341 (92.63%)	20689 (91.14%)	8952 (93.12%)
	**Emg. GP Ref.**	5866 (4.93%)	3721 (4.67%)	2145 (5.47%)	1506 (6.63%)	471 (4.9%)
	**Emg. OPD**	898 (0.76%)	627 (0.79%)	271 (0.69%)	155 (0.68%)	88 (0.92%)
	**Emg. Tran.**	315 (0.26%)	231 (0.29%)	84 (0.21%)	63 (0.28%)	14 (0.15%)
	**Non-Emg. Tran.**	1180 (0.99%)	788 (0.99%)	392 (1.0%)	288 (1.27%)	88 (0.92%)
**Admission Specialty**	**A&E**	18309 (15.4%)	12838 (16.12%)	5471 (13.94%)	3795 (16.72%)	1573 (16.36%)
	**Acute Int. Med.**	89724 (75.47%)	58358 (73.27%)	31366 (79.95%)	17326 (76.32%)	7447 (77.47%)
	**Gen. Surgery**	2595 (2.18%)	2377 (2.98%)	0 (0%)	147 (0.65%)	0 (0%)
	**General Med.**	0 (0%)	0 (0%)	328 (0.84%)	181 (0.8%)	78 (0.81%)
	**Nephrology**	2715 (2.28%)	1809 (2.27%)	906 (2.31%)	571 (2.52%)	219 (2.28%)
	**Other**	4318 (3.63%)	3282 (4.12%)	926 (2.36%)	681 (3.0%)	228 (2.37%)
	**Trauma & Orth.**	1225 (1.03%)	989 (1.24%)	236 (0.6%)	0 (0%)	68 (0.71%)
**Breathing Device**	**A - Air**	106730 (89.78%)	71828 (90.18%)	34902 (88.96%)	20447 (90.07%)	8560 (89.05%)
	**Nas. cannula**	8863 (7.46%)	5508 (6.91%)	3355 (8.55%)	1666 (7.34%)	821 (8.54%)
	**Other**	0 (0%)	0 (0%)	0 (0%)	192 (0.85%)	0 (0%)
	**Res. mask**	393 (0.33%)	0 (0%)	172 (0.44%)	107 (0.47%)	0 (0%)
	**Simple mask**	677 (0.57%)	301 (0.38%)	376 (0.96%)	247 (1.09%)	29 (0.3%)
	**Venturi 28%**	391 (0.33%)	349 (0.44%)	0 (0%)	0 (0%)	0 (0%)
**ED Diagnosis**	**Other**	1402 (1.18%)	1217 (1.53%)	1 (0.0%)	0 (0%)	0 (0%)
	**collaps-**	1245 (1.05%)	1245 (1.56%)	0 (0%)	0 (0%)	0 (0%)
	**confus**	0 (0%)	356 (0.45%)	0 (0%)	0 (0%)	0 (0%)
	**cope**	590 (0.5%)	0 (0%)	244 (0.62%)	150 (0.66%)	66 (0.69%)
	**dementia**	0 (0%)	0 (0%)	142 (0.36%)	80 (0.35%)	28 (0.29%)
	**fall**	1461 (1.23%)	1461 (1.83%)	0 (0%)	0 (0%)	0 (0%)
	**head**	2703 (2.27%)	1987 (2.49%)	716 (1.82%)	440 (1.94%)	194 (2.02%)
	**pain**	7162 (6.02%)	7139 (8.96%)	23 (0.06%)	9 (0.04%)	4 (0.04%)
	**vomit**	0 (0%)	0 (0%)	32 (0.08%)	13 (0.06%)	1 (0.01%)
**Presenting Complaint**	**Other**	36810 (30.96%)	25992 (32.63%)	10818 (27.57%)	6811 (30.0%)	2773 (28.85%)
	**abd. pain**	6411 (5.39%)	4499 (5.65%)	1912 (4.87%)	1038 (4.57%)	443 (4.61%)
	**chest pain**	11583 (9.74%)	7612 (9.56%)	3971 (10.12%)	2382 (10.49%)	990 (10.3%)
	**falls**	5277 (4.44%)	3412 (4.28%)	1865 (4.75%)	962 (4.24%)	416 (4.33%)
	**other**	31475 (26.47%)	21477 (26.96%)	9998 (25.48%)	5499 (24.22%)	2448 (25.47%)
	**short. breath**	14863 (12.5%)	9313 (11.69%)	5550 (14.15%)	2692 (11.86%)	1336 (13.9%)

Table 4. AUROC (95% bootstrapped confidence interval) of each classifier type trained on each feature set. 'Valid. (Complete)' indicates the full validation set, 'Valid. (Unseen)' the validation set excluding all patients that had admission records in the training set, and 'Valid. (Pre-Covid)' the subset of validation records that occurred before March 1st, 2020.

Features	Estimator	Valid. (Complete)	Valid. (Unseen)	Valid. (Pre-Covid)
**Reference**	**NEWS2**	0.7956 (0.7771-0.8103)	0.8098 (0.7877-0.8312)	0.7672 (0.7263-0.7980)
**Vitals**	**LR**	0.8082 (0.7886-0.8238)	0.8199 (0.8035-0.8411)	0.8000 (0.7690-0.8265)
	**LR-EN**	0.8071 (0.7874-0.8230)	0.8190 (0.8026-0.8403)	0.7999 (0.7697-0.8272)
	**LR-L1**	0.8082 (0.7886-0.8238)	0.8199 (0.8034-0.8410)	0.8002 (0.7693-0.8267)
	**LR-L2**	0.8081 (0.7886-0.8237)	0.8199 (0.8035-0.8410)	0.8000 (0.7690-0.8265)
	**LightGBM**	0.8336 (0.8154-0.8488)	0.8435 (0.8290-0.8589)	0.8257 (0.7941-0.8495)
	**LinearSVM**	0.7974 (0.7783-0.8125)	0.8079 (0.7884-0.8297)	0.7869 (0.7506-0.8187)
	**XGBoost**	0.8348 (0.8175-0.8514)	0.8433 (0.8287-0.8616)	0.8271 (0.7936-0.8525)
**& Obs**	**LR**	0.8366 (0.8189-0.8476)	0.8450 (0.8284-0.8608)	0.8285 (0.8023-0.8552)
	**LR-EN**	0.8366 (0.8188-0.8475)	0.8447 (0.8281-0.8609)	0.8283 (0.8018-0.8553)
	**LR-L1**	0.8366 (0.8188-0.8476)	0.8448 (0.8283-0.8610)	0.8281 (0.8013-0.8553)
	**LR-L2**	0.8366 (0.8188-0.8476)	0.8448 (0.8283-0.8609)	0.8284 (0.8020-0.8554)
	**LightGBM**	0.8620 (0.8494-0.8744)	0.8702 (0.8571-0.8873)	0.8605 (0.8325-0.8831)
	**LinearSVM**	0.8125 (0.7938-0.8299)	0.8157 (0.7949-0.8349)	0.8262 (0.8028-0.8518)
	**XGBoost**	0.8652 (0.8515-0.8763)	0.8770 (0.8664-0.8920)	0.8557 (0.8270-0.8797)
**& Labs**	**LR**	0.8449 (0.8286-0.8572)	0.8515 (0.8363-0.8673)	0.8291 (0.8033-0.8605)
	**LR-EN**	0.8449 (0.8283-0.8569)	0.8514 (0.8358-0.8666)	0.8291 (0.8033-0.8601)
	**LR-L1**	0.8489 (0.8335-0.8623)	0.8562 (0.8416-0.8708)	0.8384 (0.8147-0.8673)
	**LR-L2**	0.8449 (0.8284-0.8571)	0.8515 (0.8363-0.8673)	0.8290 (0.8032-0.8604)
	**LightGBM**	0.8842 (0.8724-0.8940)	0.8931 (0.8816-0.9064)	0.8676 (0.8401-0.8913)
	**LinearSVM**	0.8388 (0.8238-0.8514)	0.8457 (0.8281-0.8636)	0.8383 (0.8122-0.8723)
	**XGBoost**	0.8778 (0.8648-0.8886)	0.8865 (0.8742-0.9023)	0.8575 (0.8320-0.8854)
**& Notes**	**LR**	0.8530 (0.8380-0.8650)	0.8573 (0.8419-0.8718)	0.8422 (0.8179-0.8716)
	**LR-EN**	0.8554 (0.8394-0.8669)	0.8586 (0.8422-0.8736)	0.8448 (0.8202-0.8743)
	**LR-L1**	0.8578 (0.8429-0.8699)	0.8608 (0.8455-0.8739)	0.8484 (0.8247-0.8767)
	**LR-L2**	0.8553 (0.8394-0.8671)	0.8583 (0.8423-0.8728)	0.8451 (0.8206-0.8744)
	**LightGBM**	0.8884 (0.8755-0.8981)	0.8949 (0.8814-0.9095)	0.8733 (0.8476-0.8994)
	**LinearSVM**	0.8460 (0.8337-0.8585)	0.8481 (0.8350-0.8618)	0.8288 (0.8037-0.8614)
	**XGBoost**	0.8741 (0.8617-0.8855)	0.8849 (0.8735-0.8987)	0.8477 (0.8211-0.8777)
**& Services**	**LR**	0.8959 (0.8828-0.9063)	0.8973 (0.8866-0.9117)	0.9023 (0.8817-0.9246)
	**LR-EN**	0.8988 (0.8849-0.9102)	0.9003 (0.8881-0.9136)	0.9027 (0.8815-0.9253)
	**LR-L1**	0.9009 (0.8875-0.9119)	0.9031 (0.8914-0.9167)	0.9035 (0.8840-0.9247)
	**LR-L2**	0.8992 (0.8854-0.9106)	0.9010 (0.8892-0.9141)	0.9030 (0.8817-0.9255)
	**LightGBM**	0.9200 (0.9084-0.9295)	0.9214 (0.9115-0.9350)	0.9160 (0.8981-0.9341)
	**LinearSVM**	0.8962 (0.8840-0.9084)	0.8968 (0.8845-0.9112)	0.8897 (0.8705-0.9139)
	**XGBoost**	0.9120 (0.8989-0.9223)	0.9190 (0.9105-0.9329)	0.9052 (0.8845-0.9250)

Table 5. Average Precision (95% bootstrapped confidence interval) of each classifier type trained on each feature set. 'Valid. (Complete)' indicates the full validation set, 'Valid. (Unseen)' the validation set excluding all patients that had admission records in the training set, and 'Valid. (Pre-Covid)' the subset of validation records that occurred before March 1st, 2020.

Features	Estimator	Valid. (Complete)	Valid. (Unseen)	Valid. (Pre-Covid)
**Reference**	**NEWS2**	0.1472 (0.1278-0.1726)	0.1670 (0.1403-0.1924)	0.1511 (0.1089-0.1925)
	**LR**	0.1567 (0.1375-0.1803)	0.1724 (0.1449-0.2017)	0.1632 (0.1260-0.2039)
	**LR-EN**	0.1557 (0.1366-0.1796)	0.1702 (0.1423-0.1992)	0.1627 (0.1253-0.2059)
	**LR-L1**	0.1566 (0.1374-0.1801)	0.1722 (0.1447-0.2015)	0.1634 (0.1261-0.2041)
**Vitals**	**LR-L2**	0.1565 (0.1372-0.1801)	0.1720 (0.1446-0.2015)	0.1633 (0.1261-0.2040)
	**LightGBM**	0.1734 (0.1499-0.1963)	0.1978 (0.1694-0.2270)	0.1937 (0.1479-0.2470)
	**LinearSVM**	0.1347 (0.1178-0.1567)	0.1477 (0.1186-0.1745)	0.1574 (0.1200-0.1954)
	**XGBoost**	0.1671 (0.1439-0.1885)	0.1891 (0.1586-0.2175)	0.2004 (0.1572-0.2517)
	**LR**	0.2585 (0.2284-0.2900)	0.3100 (0.2703-0.3462)	0.2606 (0.2012-0.3160)
	**LR-EN**	0.2584 (0.2293-0.2897)	0.3103 (0.2702-0.3459)	0.2605 (0.2019-0.3147)
	**LR-L1**	0.2572 (0.2278-0.2881)	0.3096 (0.2704-0.3457)	0.2596 (0.2006-0.3142)
**& Obs**	**LR-L2**	0.2586 (0.2292-0.2895)	0.3108 (0.2710-0.3463)	0.2617 (0.2025-0.3159)
	**LightGBM**	0.3109 (0.2761-0.3483)	0.3708 (0.3246-0.4086)	0.3216 (0.2684-0.3851)
	**LinearSVM**	0.1955 (0.1716-0.2235)	0.2281 (0.1905-0.2621)	0.2102 (0.1659-0.2694)
	**XGBoost**	0.3001 (0.2670-0.3383)	0.3592 (0.3178-0.3969)	0.3189 (0.2630-0.3786)
	**LR**	0.2597 (0.2340-0.2887)	0.3020 (0.2589-0.3364)	0.2567 (0.2035-0.3105)
	**LR-EN**	0.2575 (0.2318-0.2882)	0.2997 (0.2587-0.3343)	0.2564 (0.2031-0.3112)
	**LR-L1**	0.2381 (0.2139-0.2681)	0.2774 (0.2382-0.3093)	0.2459 (0.1944-0.3017)
**& Labs**	**LR-L2**	0.2600 (0.2342-0.2895)	0.3024 (0.2599-0.3368)	0.2569 (0.2029-0.3111)
	**LightGBM**	0.3136 (0.2771-0.3544)	0.3637 (0.3233-0.3994)	0.3181 (0.2670-0.3775)
	**LinearSVM**	0.2269 (0.2043-0.2567)	0.2576 (0.2159-0.2918)	0.2208 (0.1729-0.2731)
	**XGBoost**	0.3027 (0.2674-0.3410)	0.3643 (0.3275-0.4015)	0.3103 (0.2598-0.3703)
	**LR**	0.2641 (0.2383-0.2940)	0.3093 (0.2679-0.3473)	0.2658 (0.2182-0.3211)
	**LR-EN**	0.2647 (0.2389-0.2929)	0.3106 (0.2682-0.3486)	0.2669 (0.2192-0.3224)
	**LR-L1**	0.2366 (0.2131-0.2683)	0.2773 (0.2362-0.3125)	0.2556 (0.2094-0.3183)
**& Notes**	**LR-L2**	0.2642 (0.2389-0.2927)	0.3108 (0.2685-0.3494)	0.2660 (0.2181-0.3219)
	**LightGBM**	0.3257 (0.2881-0.3619)	0.3735 (0.3347-0.4140)	0.3551 (0.2957-0.4186)
	**LinearSVM**	0.2546 (0.2262-0.2854)	0.2796 (0.2118-0.3276)	0.2547 (0.2300-0.2834)
	**XGBoost**	0.2897 (0.2632-0.3232)	0.3463 (0.3111-0.3874)	0.2871 (0.2358-0.3385)
	**LR**	0.4748 (0.4343-0.5150)	0.4846 (0.4483-0.5288)	0.5337 (0.4834-0.5912)
	**LR-EN**	0.4802 (0.4394-0.5200)	0.4890 (0.4489-0.5332)	0.5365 (0.4841-0.5938)
	**LR-L1**	0.4739 (0.4344-0.5153)	0.4802 (0.4398-0.5257)	0.5322 (0.4780-0.5940)
**& Services**	**LR-L2**	0.4798 (0.4386-0.5203)	0.4889 (0.4488-0.5328)	0.5354 (0.4832-0.5918)
	**LightGBM**	0.5133 (0.4685-0.5487)	0.5245 (0.4780-0.5671)	0.5711 (0.5260-0.6234)
	**LinearSVM**	0.3950 (0.3517-0.4310)	0.4102 (0.3662-0.4535)	0.4568 (0.4050-0.5217)
	**XGBoost**	0.4395 (0.4002-0.4739)	0.4567 (0.4165-0.4955)	0.4688 (0.4091-0.5371)

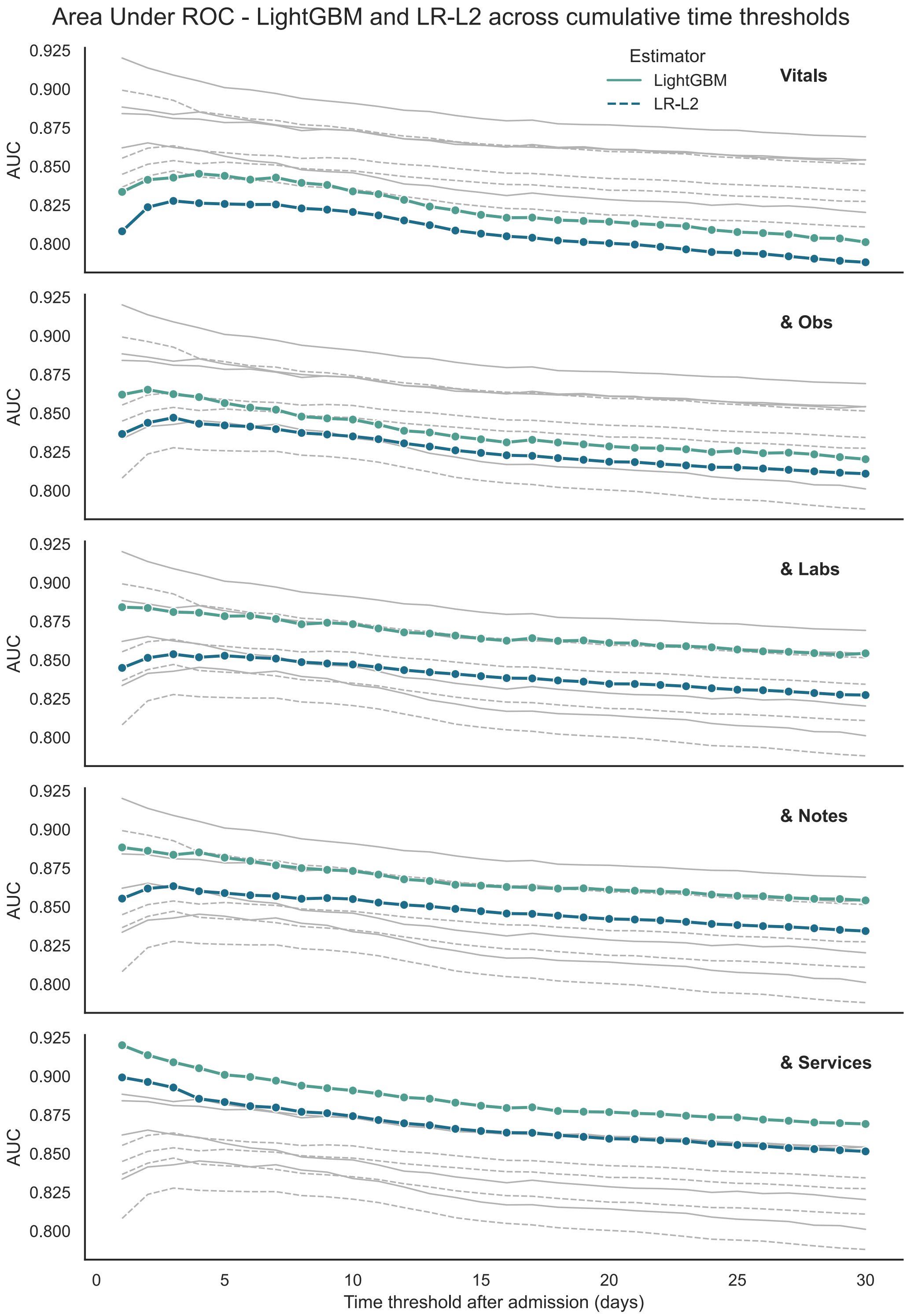

Figure 4. AUROC of logistic regression (L2 penalty) and LightGBM across thresholds for labelling critical deterioration events. Each section **(a-e)** corresponds to incrementally including the indicated feature sets (from Methods Table 4) as training data. For each one, and for each x-axis value, we train independent models to identify critical deterioration up to the corresponding number of days after admission and measure their AUROC on the validation set (y-axis). The lines in colour represent the performance for the indicated feature set, and the lines in gray represent the lines from the other sections for easier visual comparison.

Table 6. Coefficients of numerical features in logistic regression models. LR: Logistic regression; LR-L1: LR with L1 penalty regularisation; LR-L2: LR with L2 penalty regularisation; LR-EN: LR with Elastic Net regularisation.

	LR	LR-L1	LR-L2	LR-EN
**SDEC**	−1.0373	−1.0352	−1.0408	−1.0398
**Sodium (mmol/L)**	−0.8101	−0.7439	−0.804	−0.8098
**Age (years)**	−0.5275	−0.4232	−0.5256	−0.525
**FiO2 (%)**	−0.4559	−0.4474	−0.4531	−0.4548
**SpO2 (%)**	−0.2009	−0.2003	−0.2009	−0.2008
**Creatinine (mmol/L)**	−0.1947	−0.186	−0.193	−0.1928
**Assisted Breathing**	−0.1308	−0.1248	−0.1298	−0.1293
**Readmission**	−0.1051	−0.0608	−0.1049	−0.1051
**Temperature (oC)**	−0.0509	−0.0433	−0.0502	−0.0504
**Alert (AVCPU)**	−0.0312	−0.0109	−0.0262	−0.0269
**Urea (mmol/L)**	−0.0292	−0.0288	−0.03	−0.0304
**Systolic BP (mmHg)**	−0.0186	−0.02	−0.0194	−0.0197
**Pain**	0.0608	0.0643	0.0633	0.0626
**Nausea**	0.0676	0.0207	0.0684	0.0672
**Diastolic BP (mmHg)**	0.1145	0.0772	0.1102	0.1121
**Pulse (beats/min)**	0.1159	0.1152	0.1157	0.1156
**Haemoglobin (g/L)**	0.1359	0.0919	0.1341	0.134
**Vomiting**	0.1862	0.1915	0.1872	0.187
**RR (breaths/min)**	0.1974	0.1992	0.1967	0.1972
**Female**	0.2827	0.2669	0.2827	0.2835
**Lying Down**	0.3498	0.3305	0.349	0.3496
**Potassium (mEg/L)**	1.1462	0.4636	1.4898	0.7238

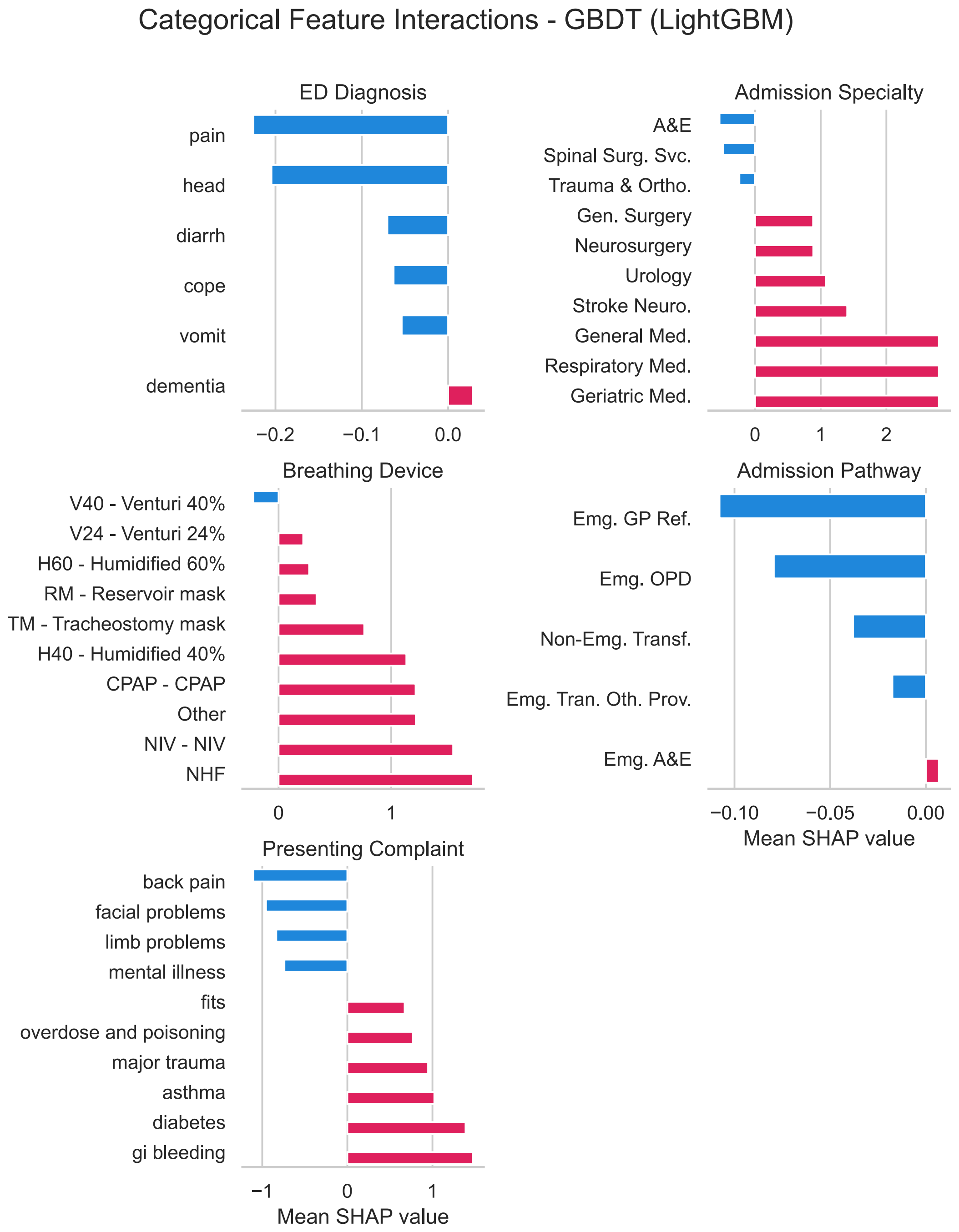

Figure 5. SHAP feature importances for categorical feature values under the LightGBM model. Each bar shows the mean SHAP value for the indicated value of the corresponding categorical feature. Values to the right of $x = 0$ (red) contribute, on average, towards predictions of deterioration, while values to the left (blue) contribute towards negative predictions (no deterioration). We provide the highest-ranked values per feature by mean absolute SHAP value.

Table 7. Coefficients of categorical feature values in logistic regression models. We provide the 5 highest ranked values per feature by mean absolute coefficient over the presented models. LR: Logistic regression; LR-L1: LR with L1 penalty regularisation; LR-L2: LR with L2 penalty regularisation; LR-EN: LR with Elastic Net regularisation.

Feature	Value	LR	LR-EN	LR-L1	LR-L2
**Admission Pathway**	**Emg. OPD**	−2.5852	−1.7068	0.0	−1.1932
	**Emg. GP Ref.**	−2.0966	−1.3112	−0.2785	−0.9036
	**Emg. Tran.**	0.3564	1.0585	0.5515	1.3829
	**Emg. A&E**	−0.4055	0.3399	0.579	0.7215
	**Non-Emg. Tran.**	−1.1169	−0.3635	0.0	−0.0228
**Admission Specialty**	**General Med.**	6.0344	4.453	4.5126	4.2537
	**Respiratory Med.**	5.7329	4.1241	3.7031	3.8324
	**Geriatric Med.**	5.1331	3.4959	2.369	3.1533
	**Bariatric Surg.**	8.2416	2.1229	0.0	1.6813
	**Spinal Surg. Svc.**	−6.0434	−1.9752	0.0	−1.5621
**Breathing Device**	**NHF**	3.1472	3.2046	3.0634	3.0471
	**Unknown**	−5.741	−1.1492	0.0	−0.6422
	**Air**	−1.253	−1.5743	−1.3892	−0.8627
	**NIV**	1.1215	1.2039	1.162	1.1301
	**Other**	0.8889	0.9771	1.1688	0.9087
**ED Diagnosis**	**dementia**	−5.5677	−1.0698	0.0	−0.8859
	**dizz-**	−4.3817	−0.4509	0.0	−0.5055
	**Unknown**	1.3982	0.5585	0.4768	0.7012
	**cope**	−1.2838	−0.5788	0.0	−0.3291
	**diarrh**	1.0958	0.2473	0.0	0.3656
**Presenting Complaint**	**ear problems**	−6.0118	−1.4829	0.0	−1.2246
	**facial problems**	−5.5813	−1.3773	0.0	−1.1297
	**gi bleeding**	2.8215	1.7377	1.2848	1.7212
	**diabetes**	2.7244	1.6404	1.1291	1.6234
	**back pain**	−1.1915	−2.1741	−1.1507	−1.9533

Table 8. Differential Fairness Bias Amplification (95% bootstrapped confidence interval) of each classifier type trained on each feature set. The columns "Sex", "Age", and "Sex & Age" indicate the protected characteristic for each measurement - biological sex, age group (per Figure 1), or both.

Features	Estimator	Sex	Age	Sex & Age
Vitals	LR	-0.249 (-0.316 - -0.151)	0.094 (-0.010 - 0.216)	-0.338 (-0.634 - -0.266)
	LR-EN	-0.249 (-0.317 - -0.149)	0.098 (-0.006 - 0.218)	-0.339 (-0.633 - -0.263)
	LR-L1	-0.249 (-0.316 - -0.151)	0.094 (-0.010 - 0.216)	-0.338 (-0.633 - -0.266)
	LR-L2	-0.249 (-0.316 - -0.151)	0.094 (-0.011 - 0.216)	-0.338 (-0.634 - -0.266)
	LightGBM	-0.251 (-0.312 - -0.145)	0.051 (-0.083 - 0.179)	-0.414 (-0.740 - -0.322)
	LinearSVM	-0.252 (-0.317 - -0.172)	0.005 (-0.110 - 0.125)	-0.475 (-0.798 - -0.441)
	XGBoost	-0.245 (-0.310 - -0.148)	0.109 (-0.016 - 0.205)	-0.329 (-0.639 - -0.233)
& Obs	LR	0.028 (-0.016 - 0.085)	-0.163 (-0.272 - 0.032)	-0.537 (-0.897 - -0.385)
	LR-EN	0.027 (-0.017 - 0.085)	-0.166 (-0.271 - 0.027)	-0.539 (-0.898 - -0.391)
	LR-L1	0.026 (-0.019 - 0.083)	-0.166 (-0.271 - 0.028)	-0.542 (-0.901 - -0.394)
	LR-L2	0.027 (-0.018 - 0.085)	-0.164 (-0.271 - 0.031)	-0.539 (-0.898 - -0.389)
	LightGBM	0.004 (-0.051 - 0.051)	-0.197 (-0.409 - -0.021)	-0.635 (-0.966 - -0.594)
	LinearSVM	-0.099 (-0.147 - -0.036)	-0.337 (-0.457 - -0.124)	-0.778 (-1.122 - -0.694)
	XGBoost	-0.031 (-0.086 - 0.017)	-0.256 (-0.400 - -0.109)	-0.585 (-0.941 - -0.516)
& Labs	LR	0.072 (0.024 - 0.135)	-0.315 (-0.428 - -0.143)	-0.605 (-0.959 - -0.478)
	LR-EN	0.070 (0.021 - 0.134)	-0.311 (-0.425 - -0.137)	-0.608 (-0.961 - -0.482)
	LR-L1	0.092 (0.045 - 0.159)	-0.393 (-0.506 - -0.218)	-0.659 (-1.006 - -0.536)
	LR-L2	0.072 (0.024 - 0.136)	-0.313 (-0.427 - -0.140)	-0.604 (-0.958 - -0.477)
	LightGBM	0.011 (-0.059 - 0.064)	-0.234 (-0.406 - -0.077)	-0.594 (-0.928 - -0.552)
	LinearSVM	0.116 (0.056 - 0.178)	-0.521 (-0.632 - -0.273)	-0.862 (-1.256 - -0.684)
	XGBoost	-0.027 (-0.088 - 0.028)	-0.229 (-0.365 - -0.098)	-0.621 (-0.934 - -0.536)
& Notes	LR	0.059 (0.002 - 0.125)	-0.189 (-0.306 - -0.013)	-0.599 (-0.940 - -0.502)
	LR-EN	0.064 (0.008 - 0.127)	-0.198 (-0.315 - -0.022)	-0.606 (-0.952 - -0.512)
	LR-L1	0.067 (0.014 - 0.136)	-0.318 (-0.449 - -0.155)	-0.683 (-1.018 - -0.595)
	LR-L2	0.062 (0.006 - 0.126)	-0.196 (-0.311 - -0.021)	-0.604 (-0.948 - -0.509)
	LightGBM	-0.021 (-0.088 - 0.033)	-0.171 (-0.324 - -0.063)	-0.587 (-0.902 - -0.563)
	LinearSVM	0.112 (0.051 - 0.189)	-0.395 (-0.511 - -0.199)	-0.831 (-1.165 - -0.733)
	XGBoost	-0.067 (-0.129 - -0.013)	-0.067 (-0.194 - 0.076)	-0.501 (-0.827 - -0.424)
& Services	LR	0.038 (-0.012 - 0.093)	-0.228 (-0.370 - -0.075)	-0.657 (-0.989 - -0.598)
	LR-EN	0.045 (-0.002 - 0.098)	-0.236 (-0.378 - -0.081)	-0.659 (-0.992 - -0.599)
	LR-L1	0.039 (-0.006 - 0.098)	-0.242 (-0.364 - -0.075)	-0.635 (-0.966 - -0.563)
	LR-L2	0.046 (-0.002 - 0.098)	-0.236 (-0.379 - -0.080)	-0.660 (-0.994 - -0.600)
	LightGBM	-0.002 (-0.070 - 0.045)	-0.209 (-0.414 - -0.086)	-0.600 (-0.911 - -0.562)
	LinearSVM	-0.016 (-0.074 - 0.042)	-0.027 (-0.130 - 0.133)	-0.509 (-0.830 - -0.396)
	XGBoost	0.001 (-0.069 - 0.060)	-0.186 (-0.348 - -0.080)	-0.543 (-0.875 - -0.503)

Table 9. Summary of model performance compared to the NEWS2 across the tested feature sets. Sensitivity, specificity, positive predictive value (PPV), negative predictive value (NPV), accuracy, F2 score, and numbers needed to evaluate (NNE) of NEWS2, GBDT (LightGBM) and logistic regression with L2 penalty (LR-L2). We fix the sensitivity of the models at three levels (0.602, 0.396, and 0.220) that match the observed sensitivity of NEWS2 at thresholds 3, 5, and 7, respectively.

Features	Estimator	Threshold	Sensitivity	Specificity	PPV	NPV	Accuracy	F2	NNE
**Reference**	**NEWS2**	≥ 3	0.6021	0.8545	0.0865	0.9895	0.8489	0.2746	11.5663
		≥ 5	0.3968	0.9590	0.1813	0.9858	0.9465	0.3206	5.5144
		≥ 7	0.2201	0.9867	0.2749	0.9822	0.9696	0.2292	3.6373
**Vitals**	**LightGBM**	≥ 0.061	0.6021	0.9010	0.1221	0.9900	0.8943	0.3370	8.1932
		≥ 0.121	0.3968	0.9614	0.1902	0.9859	0.9487	0.3260	5.2586
		≥ 0.238	0.2189	0.9869	0.2767	0.9822	0.9697	0.2285	3.6146
	**LR-L2**	≥ 0.056	0.5998	0.8904	0.1112	0.9898	0.8839	0.3193	8.9924
		≥ 0.110	0.3979	0.9485	0.1501	0.9857	0.9362	0.2992	6.6619
		≥ 0.207	0.2201	0.9844	0.2440	0.9822	0.9673	0.2245	4.0984
**& Obs**	**LightGBM**	≥ 0.075	0.6021	0.9252	0.1555	0.9903	0.9180	0.3824	6.4318
		≥ 0.181	0.3968	0.9801	0.3127	0.9861	0.9670	0.3765	3.1983
		≥ 0.354	0.2178	0.9951	0.5053	0.9823	0.9777	0.2458	1.9791
	**LR-L2**	≥ 0.051	0.6043	0.8935	0.1149	0.9900	0.8871	0.3263	8.7038
		≥ 0.149	0.3934	0.9683	0.2209	0.9859	0.9554	0.3402	4.5275
		≥ 0.297	0.2201	0.9936	0.4416	0.9824	0.9763	0.2446	2.2642
**& Labs**	**LightGBM**	≥ 0.106	0.6021	0.9416	0.1908	0.9904	0.9340	0.4207	5.2405
		≥ 0.230	0.3968	0.9834	0.3537	0.9862	0.9703	0.3874	2.8276
		≥ 0.360	0.2201	0.9946	0.4825	0.9824	0.9773	0.2469	2.0725
	**LR-L2**	≥ 0.059	0.6021	0.9052	0.1267	0.9900	0.8984	0.3440	7.8902
		≥ 0.158	0.3740	0.9752	0.2562	0.9855	0.9617	0.3425	3.9024
		≥ 0.317	0.2201	0.9936	0.4386	0.9824	0.9763	0.2444	2.2798
**& Notes**	**LightGBM**	≥ 0.094	0.6021	0.9442	0.1978	0.9905	0.9365	0.4274	5.0549
		≥ 0.205	0.3945	0.9825	0.3402	0.9861	0.9694	0.3823	2.9393
		≥ 0.336	0.2201	0.9950	0.5000	0.9824	0.9776	0.2478	2.0000
	**LR-L2**	≥ 0.060	0.5986	0.9081	0.1297	0.9900	0.9012	0.3474	7.7105
		≥ 0.158	0.3945	0.9718	0.2421	0.9860	0.9589	0.3504	4.1301
		≥ 0.311	0.2121	0.9945	0.4685	0.9822	0.9770	0.2382	2.1344
**& Services**	**LightGBM**	≥ 0.167	0.6021	0.9735	0.3417	0.9907	0.9652	0.5225	2.9261
		≥ 0.432	0.3957	0.9949	0.6379	0.9863	0.9815	0.4282	1.5677
		≥ 0.810	0.2189	0.9996	0.9231	0.9824	0.9821	0.2583	1.0833
	**LR-L2**	≥ 0.114	0.6009	0.9633	0.2726	0.9906	0.9552	0.4843	3.6679
		≥ 0.360	0.3968	0.9946	0.6259	0.9863	0.9812	0.4281	1.5977
		≥ 0.788	0.2155	0.9993	0.8832	0.9824	0.9818	0.2539	1.1323

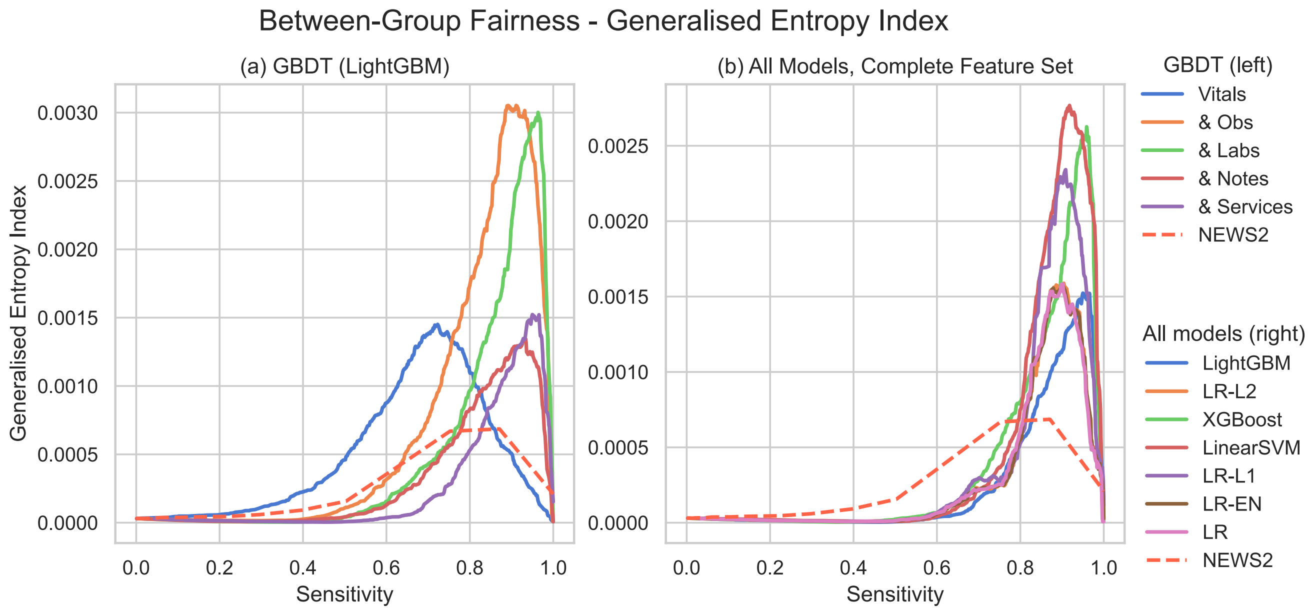

Figure 6. Between-Group Generalised Entropy vs Sensitivity curves of **(a)**: LightGBM across the tested feature sets, and **(b)**: All classifier types trained on the complete feature set. We plot the between-group component of the generalised entropy index, representing unfairness between demographic groups defined by the protected characteristics of age group and sex, per Figure 1. The remainder of the generalised entropy, as presented in Figure 6 is the *within-group* component, representing all other potential biases. A lower value on the y-axis indicates a more fair distribution of 'benefit', i.e. of receiving a positive prediction, between the demographic groups we consider. A theoretical 'perfect' model would yield a single point $(0,1)$ in the lower-right corner of the plot.

Table 10. TRIPOD Checklist: Prediction Model Development

Section/Topic	Item	Checklist Item	Page
**Title and abstract**			
Title	1	Identify the study as developing and/or validating a multivariable prediction model, the target population, and the outcome to be predicted.	1
Abstract	2	Provide a summary of objectives, study design, setting, participants, sample size, predictors, outcome, statistical analysis, results, and conclusions.	1
**Introduction**			
Background and objectives	3a	Explain the medical context (including whether diagnostic or prognostic) and rationale for developing or validating the multivariable prediction model, including references to existing models.	1-2
	3b	Specify the objectives, including whether the study describes the development or validation of the model or both.	2
**Methods**			
Source of data	4a	Describe the study design or source of data (e.g., randomized trial, cohort, or registry data), separately for the development and validation data sets, if applicable.	12
	4b	Specify the key study dates, including start of accrual; end of accrual; and, if applicable, end of follow-up.	12
Participants	5a	Specify key elements of the study setting (e.g., primary care, secondary care, general population) including number and location of centres.	12
	5b	Describe eligibility criteria for participants.	12; Supplementary Figure 2
	5c	Give details of treatments received, if relevant.	N/A
Outcome	6a	Clearly define the outcome that is predicted by the prediction model, including how and when assessed.	13
	6b	Report any actions to blind assessment of the outcome to be predicted.	N/A
Predictors	7a	Clearly define all predictors used in developing or validating the multivariable prediction model, including how and when they were measured.	14; Table 4
	7b	Report any actions to blind assessment of predictors for the outcome and other predictors.	N/A
Sample size	8	Explain how the study size was arrived at.	12; Supplementary Figure 2
Missing data	9	Describe how missing data were handled (e.g., complete-case analysis, single imputation, multiple imputation) with details of any imputation method.	14
Statistical analysis methods	10a	Describe how predictors were handled in the analyses.	14
	10b	Specify type of model, all model-building procedures (including any predictor selection), and method for internal validation.	14
	10d	Specify all measures used to assess model performance and, if relevant, to compare multiple models.	15-16
Risk groups	11	Provide details on how risk groups were created, if done.	N/A
**Results**			
Participants	13a	Describe the flow of participants through the study, including the number of participants with and without the outcome and, if applicable, a summary of the follow-up time. A diagram may be helpful.	3
	13b	Describe the characteristics of the participants (basic demographics, clinical features, available predictors), including the number of participants with missing data for predictors and outcome.	4; Table 1; Supplementary Table 3
Model development	14a	Specify the number of participants and outcome events in each analysis.	3
	14b	If done, report the unadjusted association between each candidate predictor and outcome.	N/A
Model specification	15a	Present the full prediction model to allow predictions for individuals (i.e., all regression coefficients, and model intercept or baseline survival at a given time point).	Supplementary Table 2
	15b	Explain how to the use the prediction model.	14
Model performance	16	Report performance measures (with CIs) for the prediction model.	5; Supplementary Tables 4, 5; Supplementary Figure 3
**Discussion**			
Limitations	18	Discuss any limitations of the study (such as nonrepresentative	11-12

		sample, few events per predictor, missing data).	
Interpretation	19b	Give an overall interpretation of the results, considering objectives, limitations, and results from similar studies, and other relevant evidence.	8, 11
Implications	20	Discuss the potential clinical use of the model and implications for future research.	11
**Other information**			
Supplementary information	21	Provide information about the availability of supplementary resources, such as study protocol, Web calculator, and data sets.	16
Funding	22	Give the source of funding and the role of the funders for the present study.	16
